# Regional zenith tropospheric delay prediction using DBO-optimized CNN-LSTM with multihead attention

**DOI:** 10.1038/s41598-025-15376-z

**Published:** 2025-08-12

**Authors:** Ruixue Yang, Xu Yang, Shicheng Xie, Xuexiang Yu

**Affiliations:** 1 Key Laboratory of Aviation-aerospace-ground Cooperative Monitoring and Early Warning of Coal Mining-induced Disasters of Anhui Higher Education Institutes, Anhui University of Science and Technology, KLAHEI (KLAHEI18015), Huainan, 232001 China; 2 Engineering Research Center of Mining Area Environmental and Disaster Cooperative Monitoring, Anhui University of Science and Technology, Huainan, 232001 China; 3 Urban 3D Real Scene and Intelligent Security Monitoring Joint Laboratory of Anhui Province, Huainan, 232001 China; 4 The Key Laboratory of Universities in Anhui Province for Prevention of Mine Geological Disasters, Huainan, 232001 China; 5 School of Geomatics, Anhui University of Science and Technology, Huainan, 232001 China

**Keywords:** ZTD modeling, DBO, CNN-LSTM-Multihead-Attention, Deep learning, Spatio-temporal sequence prediction, Climate sciences, Atmospheric science

## Abstract

Zenith Total Delay (ZTD) is integral to applications such as atmospheric water vapor inversion and precise positioning in the Global Navigation Satellite System (GNSS). The development of high-precision regional ZTD models has emerged as a significant area of research within the GNSS domain. This study addresses the challenges associated with achieving high-precision tropospheric delay predictions under specific conditions and the limitations of CNN-LSTM models, particularly regarding suboptimal hyperparameter optimization and convergence to local optima. We propose a novel regional ZTD prediction model, the CNN-LSTM-Multihead-Attention (CLMA) model, optimized using the Dung Beetle Optimization (DBO), referred to as ZTD-DBO-CLMA. This model synergistically integrates the spatial feature extraction capabilities of Convolutional Neural Networks (CNN) with the temporal sequence modeling strengths of Long Short-Term Memory (LSTM) networks, enhanced through advanced hyperparameter optimization techniques. The model facilitates synchronized learning of CNN and LSTM components via the DBO optimization algorithm and the incorporation of a multihead attention mechanism.In our study, we utilized five consecutive months of ZTD data from 40 International GNSS Service (IGS) stations within the European region, sampled at one-hour intervals, to investigate regional ZTD prediction models. We employed the ZTD-DBO-CLMA model and compared it to the ZTD-CLMA model, which lacks DBO optimization. The results indicate that the ZTD-DBO-CLMA model significantly enhances prediction accuracy, reducing the mean absolute error (MAE) and root mean square error (RMSE) by 0.31 mm and 1.38 mm, respectively, while increasing the coefficient of determination (R²) by 39.43%. Furthermore, the DBO algorithm consistently demonstrates its optimization efficacy across diverse weather conditions, thereby improving the precision of ZTD predictions.

## Introduction

Atmospheric water vapor serves as a pivotal information source for forecasting global climate change, precipitation, and extreme weather events. However, it also represents a significant source of error in Earth observation systems, such as the Global Navigation Satellite System (GNSS). Atmospheric modeling products are indispensable across various domains, including GNSS meteorological research, autonomous driving, aerospace, and crustal movement monitoring^1–6^. Among these products, the Zenith Total Delay (ZTD) regional modeling product is particularly important. Regional ZTD models can be categorized into two primary types based on their application requirements, specifically whether meteorological parameters are necessary. The first category comprises classical models that depend on meteorological parameter support, such as the Black, Hopfield, and Saastamoinen models. Due to geographical limitations and high costs, GNSS monitoring stations frequently fall short of the requirements for acquiring meteorological sensor data in practical navigation and positioning applications^7^leading to challenges in these areas. To overcome this limitation, researchers have devised models that operate independently of meteorological parameters, classified as the second category of models. The fundamental approach of these models entails the development of novel empirical frameworks utilizing extensive historical datasets, including the UNB series, GPT series, GZTD series, and EGNOS series. This study concentrates on the investigation of the second type of model^8^.

In recent years, the advancement of high-spatial-temporal-resolution ZTD products, coupled with the growing demand for high-precision forecasting tools in domains such as intelligent surveying and mapping, has underscored the critical importance of developing a regional high-precision ZTD model^2^. Precise Point Positioning (PPP) technology, which depends on precise satellite orbit and clock difference information, enables global absolute positioning accuracy at the centimeter or even millimeter scale^3^. Nevertheless, this method necessitates a relatively extended convergence period to attain high-precision positioning and ZTD estimation. To improve PPP, the study in literature^4^ developed a regional ZTD interpolation model to derive accurate ZTD values, which was subsequently refined into a precise point positioning real-time kinematic (PPP-RTK) model. Reference^5^ illustrated the efficacy of incorporating additional atmospheric external constraints to reduce the convergence time of precise positioning and enhance positioning accuracy. Furthermore, by integrating ground-based GNSS observation data with ERA5 meteorological data—the latest climate reanalysis dataset from the European Climate Change Mechanism’s fifth generation—a high-precision real-time ZTD model was established.

Recent advancements in deep learning technology for time series forecasting have shown considerable promise in enhancing the accuracy of ZTD predictions^9^. In a study by Reference^10^an artificial neural network (ANN) model was employed to forecast 6-hour zenith wet delay (ZWD) during wet and dry periods in Austria. However, the choice of these time periods may be somewhat arbitrary given varying weather conditions. Reference^11^ introduced a Gorilla Troops Optimizer (GTO)-enhanced ANN model (GTO-ANN) for ZWD prediction, demonstrating superior performance over both traditional and enhanced ANN models. Additionally, Reference^12^ explored the use of a Support Vector Regression (SVR) model, incorporating three kernel functions—linear, polynomial, and radial basis function (RBF)—for ZTD prediction. Results from four stations situated in diverse global regions revealed that the RBF-SVR model achieved optimal performance, remaining unaffected by variations in station altitude. This model exhibited exceptional efficacy in mid-to-high latitude regions, although its performance diminished in low-latitude areas. In Reference^13^ the Long Short-Term Memory (LSTM) model was employed to leverage its strengths in time series analysis for the modeling and prediction of tropospheric wet delay across four tropical regions globally. The model’s performance was quantified using the Root Mean Square Error (RMSE) and Mean Absolute Error (MAE), which demonstrated accuracy levels within the ranges of 18.96–21.16 cm and 14.08–16.38 cm, respectively. In Reference^14^ a comprehensive analysis of the multidimensional factors affecting ground subsidence was conducted. This study integrated GNSS tropospheric delay data with the LSTM model to forecast ground subsidence time series. The results of this research offer effective prediction strategies for ground subsidence in diverse terrains and regions where weather data coverage is limited. In Reference^15^ the authors conducted Zenith Wet Delay (ZWD) modeling for the South American region, introducing an innovative structural hybrid deep learning algorithm (Convolutional Neural Networks-LSTM, CNN-LSTM, CL) to develop ZWD models under various configurations, with intentions to extend this research to a global scale. Reference^16^ enhanced the global ZTD model by employing an improved LSTM algorithm, proposing a novel ZTD prediction model designed to improve both the accuracy of ZTD predictions and the capability for long-term forecasting. Machine learning technology is pivotal not only in the real-time prediction of ZTD and in achieving high-precision navigation and positioning with GNSS^17^ but it is also essential in GNSS meteorological research^18^. Consequently, high-precision modeling of tropospheric delay is critically important. The Dung Beetle Optimization (DBO) algorithm demonstrates exceptional capabilities. For instance, as illustrated in Reference^19^ an enhanced version of the DBO algorithm is employed to optimize the parameters of a BiLSTM model, resulting in improved predictive performance and stability. In a similar vein, the study referenced as^20^ introduced the DBO-Random Forest (DBO-RF) algorithm, which markedly enhanced the predictive accuracy of the random forest model, achieving reductions in the mean absolute error and mean squared error to 0.44321 and 0.44440, respectively. Concurrently, the literature cited as^21^ developed a hybrid deep learning model, DBO-BiLSTM, which utilized the DBO optimization algorithm to attain high-precision predictions, with root mean square errors (RMSE) of 0.57 TECU and 0.92 TECU for 1-hour and 2-hour forecasts, respectively. Collectively, these innovative ZTD models have exhibited considerable advantages in temporal and spatial sequence prediction. Nonetheless, the capacity of these models to deliver high-precision ZTD predictions under complex conditions, as well as the efficacy of the DBO-optimized CNN-LSTM model, remains insufficiently explored.

This study addresses the challenge of achieving high-precision predictions of tropospheric delay in machine learning models for intelligent surveying under specific conditions, such as the uneven distribution of GNSS stations, continuous time periods, and varying weather conditions. Given the suboptimal performance of CNN-LSTM model hyperparameter optimization and its propensity to become trapped in local optima, this paper introduces the CNN-LSTM-Multihead-Attention (ZTD-DBO-CLMA) prediction model, which is optimized using the DBO. By capitalizing on the optimization capabilities of DBO^22^ the proposed model enhances the accuracy and performance of regional ZTD prediction models, thereby facilitating high-precision predictions of tropospheric delay. Utilizing ZTD data from 40 International GNSS Service (IGS) stations in the European region over a continuous five-month period, with a one-hour sampling interval, this study conducts regional ZTD prediction model analyses using both the ZTD-DBO-CLMA model and the ZTD-CLMA model (the CLMA model without DBO optimization).

## Data and methods

### Identification of the study area

**Fig. 1:**
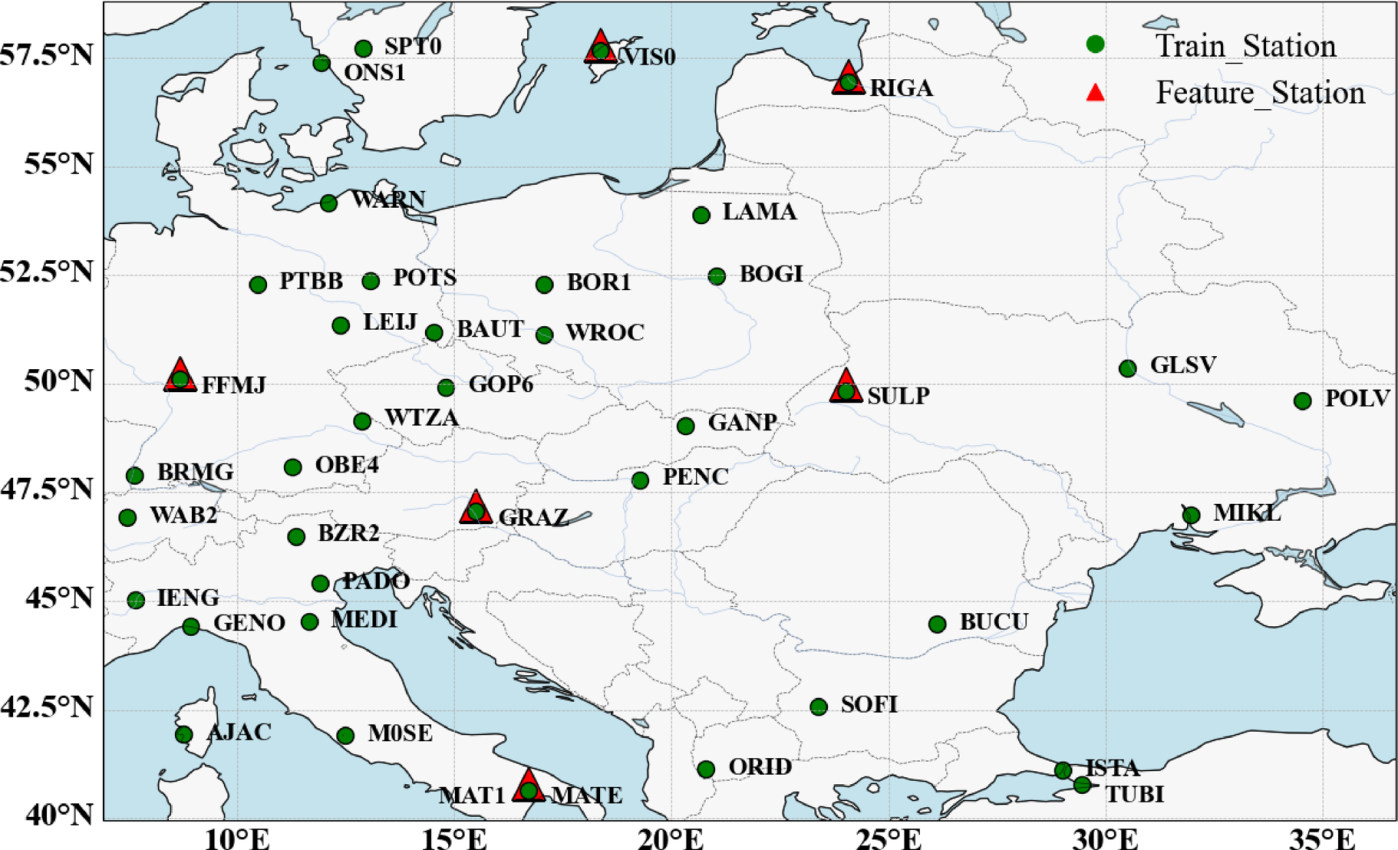
Regional site distribution map.

This study designates the coverage area of the IGS reference station network in Europe as the research region. Variations in the ZTD values within this network are intricately linked to several factors, including regional location, station elevation, climate change, and meteorological events^23^. The research region is situated in the European plain, characterized by gentle topography. However, due to land-sea contrasts, the distribution of stations within this area is non-uniform. During validation experiments, to adequately account for factors such as data volume, accuracy, stability of data sources, and reliability for data validation, 40 IGS stations were randomly selected from various locations within the study area, ensuring a minimum distance of 500 m between stations. Multiple tests were conducted to demonstrate the superior comprehensive performance of the optimized model. Furthermore, this study emphasizes six IGS stations—FFMJ, GRAZ, MATE, VIS0, SULP, and RIGA—that exhibit pronounced land-sea differences and uneven regional station distribution. This focus aims to assess the stability and robustness of the ZTD prediction model presented in this paper. Fig. 1 illustrates the distribution of regional stations. The ZTD values from 40 green circular Train_Station sites are utilized for comprehensive site modeling and predictive analysis. In contrast, the ZTD values from six red triangular Feature_Station sites are employed for modeling and predictive analysis specific to Feature_Station sites^14^.

**Table 1:** Height information for 40 IGS stations.

Site Name	Height Value	Site Name	Height Value
AJAC	50.08089	IENG	267.01680
BAUT	168.97588	ISTA	109.94775
BOGI	109.19089	LAMA	157.66258
BOR1	88.83937	LEIJ	134.21948
BRMG	212.32584	M0SE	72.18007
BUCU	107.64173	MAT1	489.01126
BZR2	281.12920	PTBB	87.21642
FFMJ	130.14323	MEDI	10.17965
GANP	703.89650	MIKL	68.32389
GENO	109.99407	OBE4	604.28560
GLSV	200.77020	ONS1	7.91701
GOP6	547.60425	SOFI	1074.45800
GRAZ	490.81055	SPT0	185.06064
ORID	730.74130	SULP	339.62753
PADO	20.08790	TUBI	182.97922
PENC	248.30635	VIS0	54.76146
POLV	159.79288	WAB2	561.73600
POTS	103.99185	WARN	12.27599
MATE	489.01126	WROC	140.55183
RIGA	13.58371	WTZA	619.08790

Table 1. Shows the elevation information for the study area sites.

### Describe the dataset

This study employs the IGS post-processed ZTD dataset, sourced from the European Centre for Medium-Range Weather Forecasts (ECMWF) website, specifically the ERA5 hourly data on pressure levels spanning from 1940 to the present. The dataset covers the period from May to September 2022, with May, July, and August each comprising 31 days, and June and September each comprising 30 days, excluding days from December 2022 to July 2023. The dataset is characterized by its high resolution and stability, making it a widely acknowledged and reliable source for regional ZTD modeling^3,8,9^. In this investigation, ZTD prediction models were comprehensively analyzed on a monthly basis. The proposed model in this paper was utilized to select the top 90% of ZTD values for each month for modeling purposes, while the bottom 10% of ZTD values were predicted. The corresponding ZTD values provided by IGS served as the true values, referred to as Real-Zenith Total Delay (R-ZTD), to assess the performance of the prediction models.

### Parallel CNN-LSTM multihead attention mechanism model

This paper seeks to undertake a comprehensive examination of regional (ZTD prediction models. Recognizing the inherent spatio-temporal variability of ZTD, it is imperative to enhance prediction accuracy by integrating both temporal and spatial dimensions. Long Short-Term Memory (LSTM) networks are adept at capturing long-term dependencies within ZTD time series data, whereas CNN excel at extracting local features in the spatial dimension^24^. Previous ZTD models have encountered challenges such as the independent modeling of temporal and spatial dimensions and the uneven distribution of observational sites, which adversely affect modeling accuracy. This study addresses these issues by concurrently considering both dimensions. By integrating CNN and LSTM in parallel, the proposed model significantly reduces the computational time required to identify optimal parameters and enhances the predictive accuracy of ZTD models. Nevertheless, the multi-head attention mechanism (MHA) offers distinct advantages^25^. The expressive capacity of the model architecture is significantly enhanced through the parallel computation of multiple sets of LSTM sub-unit attentions, thereby fully leveraging computer hardware accelerators such as GPUs to optimize the efficiency of deep learning model training and inference. In this study, multihead attention effectively addresses the challenge of long-range dependencies in the modeling of extended sequences. The fundamental aspect of the multihead mechanism technology lies in the linear projection of the query, key, and value matrices of the input sequence into multiple subspaces. Each subspace independently computes scaled dot-product attention, facilitating the capture of semantic or structural features at various levels^26^. For instance, when provided with an input embedding matrix, the multihead mechanism partitions it into H attention heads, with each head computed using the following formula:

1 $$h{\text{ead}}_{{\text{i}}} \: = \:{\text{Attention}}({\text{XW}}_{{\text{i}}}^{{\text{Q}}} ,\:{\text{XW}}_{{\text{i}}}^{{\text{K}}} ,\:{\text{XW}}_{{\text{i}}}^{{\text{V}}} )\: = {\text{soft}}\left( {\frac{{{\text{XW}}_{{\text{i}}}^{{\text{Q}}} \left( {{\text{XW}}_{{\text{i}}}^{{\text{K}}} } \right)^{{\text{T}}} }}{{\sqrt {{\text{d}}_{{\text{k}}} } }}} \right)\:{\text{XW}}_{{\text{i}}}^{{\text{v}}}$$

**Fig. 2:**
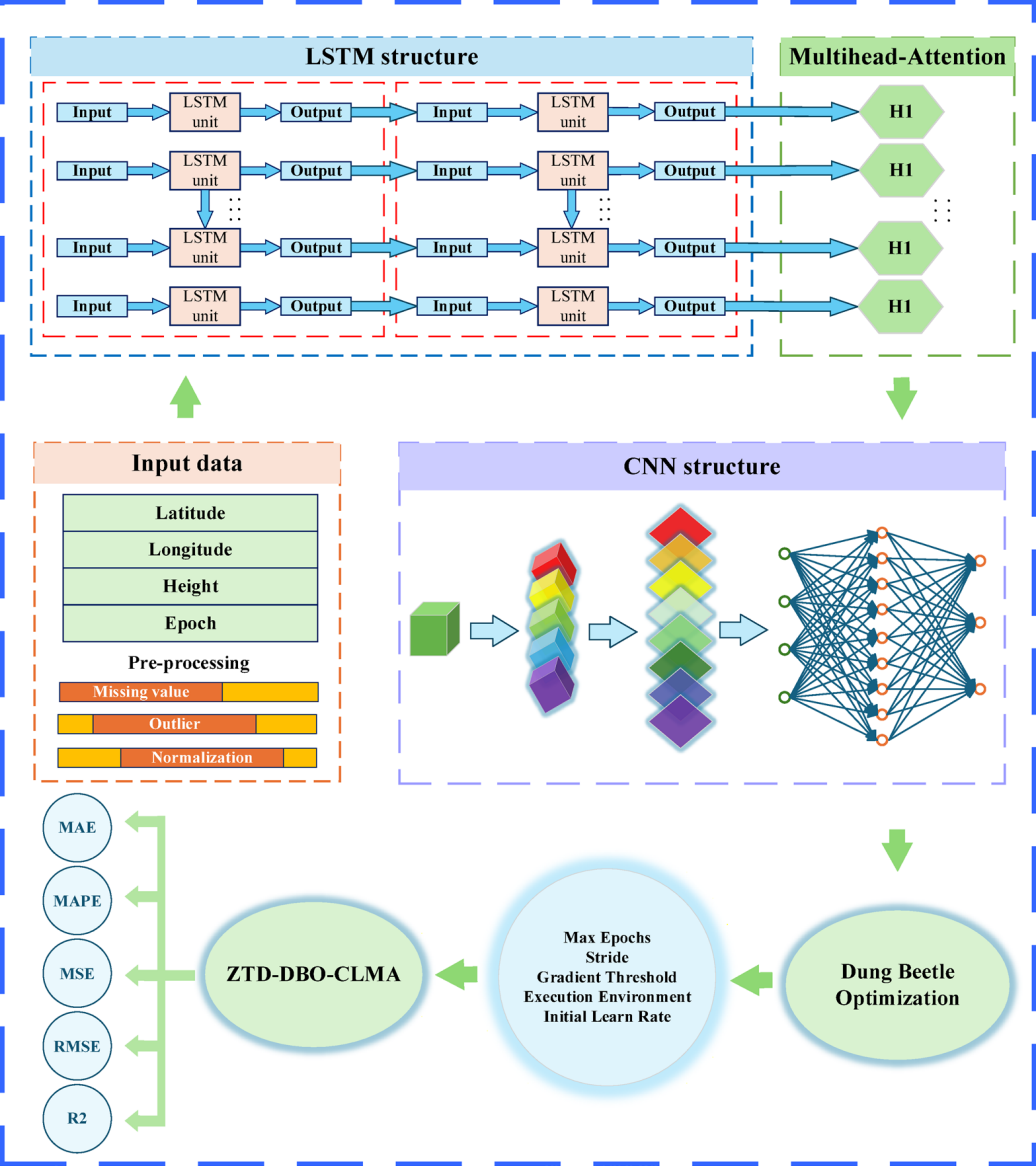
Flowchart for the Optimization Analysis of the ZTD-DBO-CNN-LSTM-Multihead-Attention Deep Learning Network.

Among these components, $\:\text{X}{\text{W}}_{\text{i}}^{\text{Q}},\:\text{X}{\text{W}}_{\text{i}}^{\text{K}},\:\text{X}{\text{W}}_{\text{i}}^{\text{V}}\:{\upepsilon\:}\:{\text{R}}^{\text{d}\times\:{\text{d}}_{\text{k}}}$ represents the learnable parameter matrix, while $\:{\text{d}}_{\text{k}}$ denotes the subspace dimension. The outputs from all heads are concatenated and subsequently integrated via the linear transformation $\:{\text{W}}^{\text{O}}$ to generate the final representation, as illustrated in Fig. 2.

2 $$\:\text{M}\text{H}\text{A}\:=\:\text{C}\text{o}\text{n}\text{c}\text{a}\text{t}({\text{h}\text{e}\text{a}\text{d}}_{1},\:...,\:{\text{h}\text{e}\text{a}\text{d}}_{\text{H}}){\text{W}}^{\text{O}}$$

In the parallel CNN-LSTM multi-head attention mechanism model, the LSTM component is responsible for modeling temporal dynamics, while the CNN is tasked with extracting local spatial features. The multi-head attention mechanism further enhances the model’s capability by capturing long sequence dependencies and facilitating multi-granularity interactions. These three components are optimized concurrently and function synergistically^27^. The model’s core value in this study is its capacity to dynamically capture critical information from the input data. Specifically, it effectively establishes long-range temporal dependencies, thereby addressing long sequence dependency challenges, and it is capable of learning multiple dependencies in parallel.

### Parallel ZTD-CLMA model for DBO optimisation

This study introduces a parallel CNN-LSTM multi-head attention mechanism model, termed ZTD-DBO-CLMA, which is optimized using the DBO algorithm. The objective is to leverage sequence data with regional spatiotemporal correlations for predicting ZTD. The ZTD-CLMA model integrates the spatial feature extraction capabilities of with the temporal sequence modeling strengths of LSTM networks, while employing a multi-head attention mechanism to emphasize salient features^28^. The adaptive dynamic parameters provided by DBO effectively enhance the optimization of the ZTD-CLMA-LSTM-Multihead-Attention (ZTD-CLMA) model parameters. Consequently, the ZTD-DBO-CLMA model exhibits superior global and local search capabilities, thereby facilitating the concurrent learning processes of CNN and LSTM during the input of ZTD data^29^. In the context of regional ZTD modeling, the input variables comprise station longitude, latitude, altitude, epoch time, and ZTD values. The preprocessing phase involves the removal of outliers, compensation for missing values, and normalization. Subsequently, the preprocessed dataset undergoes processing through a LSTM layer, with a lag time of 24 h for the ZTD values within the LSTM network, resulting in the dataset unit module H1, as depicted in Fig. 2. This is followed by entry into a multi-head attention mechanism layer, which facilitates optimization and iteration by computing information from various subspaces. Each attention head is capable of focusing on distinct segments of the input sequence, thereby not only enhancing the model’s capacity for modeling but also enabling it to capture a wider array of features. The ZTD-CLMA prediction model is developed by focusing on critical features through the multi-head attention mechanism^28^, enabling the simultaneous processing of temporal and spatial information for regional ZTD. Subsequently, the dataset is processed through the deep learning CNN structure layer, resulting in the formation of the CLMA model, which enhances the model’s capability to analyze sequence data from multiple perspectives. Finally, the DBO optimization technique is applied to the ZTD-CLMA model, yielding the ZTD-DBO-CLMA model. The performance of this model is assessed using various metrics, including Mean Absolute Error (MAE), Mean Absolute Percentage Error (MAPE), Mean Squared Error (MSE), Root Mean Squared Error (RMSE), and the Coefficient of Determination (R²).

### Hyperparameter optimisation

Hyperparameter optimization, also referred to as hyperparameter tuning, entails the configuration of variables and the adjustment of the neural network training process to enhance model performance in machine learning and deep learning algorithms^30^. Reference^31^ employed Long Short-Term Memory (LSTM) networks in conjunction with genetic algorithms to optimize hyperparameters, successfully generating ensemble forecasts of three-dimensional moist convective activity fields under varying weather conditions. Consequently, the scientific training of more efficient ZTD prediction models through hyperparameter optimization is of paramount importance^32^. This study utilizes the DBO method to optimize the ZTD-CLMA model, thereby enabling the ZTD-DBO-CLMA model to attain optimal performance within a specified timeframe and achieve the best possible model configuration. The process of parameter tuning is often challenged by high repetition rates and the difficulty of achieving global optimality, challenges that DBO effectively addresses and mitigates^33^. In this study, the DBO algorithm is utilized to emulate beetle behavior for the purpose of identifying optimal hyperparameters. It employs a memory search strategy to optimize the ZTD-CLMA. This approach significantly improves the generalization capability and predictive performance of the ZTD prediction model, particularly in scenarios where ZTD data is either unavailable or inaccurate. It effectively circumvents local optima in the global parameter settings of the model and achieves high-precision predictions through an optimized LSTM network^29^. Table 2 delineates the specific parameter values used for hyperparameter optimization.

**Table 2:** Specific parameter values for hyperparameter tuning.

Model	Hyper-parameters
Max Epochs	150
Stride	10
Gradient Threshold	1
Execution Environment	CPU
Initial Learn Rate	0.01

The maximum number of training epochs is configured to 150, signifying that the model will undergo 150 iterations over the dataset. The stride is set to 10, regulating the step size of the sliding window during convolution operations. Gradient clipping is applied with a threshold of 1 to mitigate the risk of gradient explosion. The model training is executed on a central processing unit (CPU), as specified by the execution environment. The initial learning rate is established at 0.01, dictating the magnitude of each update by the optimization algorithm, with a smaller learning rate facilitating stable convergence. The hardware specifications include an NVIDIA RTX 4060 GPU with 16GB of memory, an Intel Core i9-13900HX CPU featuring 24 cores and 32 threads with a turbo boost of up to 5.4 GHz, and 16GB of DDR5 RAM.

The optimization efficacy of the DBO algorithm is evident. Fundamentally, this algorithm emulates the diverse behaviors of dung beetles to identify optimal hyperparameters, thereby achieving superior predictive outcomes. The subsequent equation models the rolling behavior of dung beetles, serving as the foundational principle of the optimization process:

3 $$\left. \begin{gathered} x_{i} \left( {t + 1} \right) = x_{i} \left( t \right) + \alpha \times k \times x_{i} \left( {t - 1} \right) + b \times \Delta x \hfill \\ \Delta x = \left| {x_{i} \left( t \right) - X^{\omega } } \right| \hfill \\ \end{gathered} \right\}$$

where t denotes the current number of iterations, k denotes a constant value representing the deflection coefficient, and b is a constant value between (0, 1); $\alpha$is the natural coefficient, $\alpha$= 1 means no deviation, $\alpha$=-1 means deviation from the original direction; ${X^\omega }$denotes the global worst position; and $\Delta {\text{x}}$ is the simulated change in the light intensity, the larger means the weaker the light is;

When the dung beetle encounters an obstacle, it will change direction through dancing behavior. Its mathematical model is mainly expressed through the tangent function:

4 $${x_i}\left( {t+1} \right)={x_i}\left( t \right)+\tan \left( \theta \right)\left| {{x_i}\left( t \right) - {x_i}\left( {t - 1} \right)} \right|$$

$\tan \left( \theta \right)$ is the deflection system angle.

Female dung beetles select a strategy model to determine the boundary for egg-laying iterations. During the iteration process, the position of the egg ball changes dynamically. This process is called reproductive behavior:

5 $$\left\{ \begin{gathered} Lb^{*} = \max \left( {X^{*} \times \left( {1 - R} \right),Lb} \right) \hfill \\ Ub^{*} = \min \left( {X^{*} \times \left( {1 + R} \right),Ub} \right) \hfill \\ \end{gathered} \right.$$

Where, $Lb^{*}$, $Ub^{*}$ denote the lower and upper bounds of the spawning region, respectively; $X^{*}$, denote the current local optimal solution; $R = 1 - t/T\max$, $T\max$ denote the maximum number of iterations, $Lb$ and $Ub$, denote the lower and upper bounds of the optimisation problem, respectively. The region where spawning occurs is dynamically adjusted with the number of iterations. The dung beetle searching for the best foraging area is dynamically updated, and the following process simulates the foraging behaviour of the dung beetle:

6 $$\left\{ \begin{gathered} Lb^{b} = \max \left( {X^{b} \times \left( {1 - R} \right),Lb} \right) \hfill \\ Ub^{b} = \min \left( {X^{b} \times \left( {1 + R} \right),Ub} \right) \hfill \\ \end{gathered} \right.$$

where $X^{b}$, denotes the global optimal position, $Lb^{b}$ and $Ub^{b}$, denote the lower and upper bounds of the optimisation problem, respectively. $R = 1 - t/T$, $T$ is the maximum number of iterations; $Lb^{b}$ and $Ub^{b}$ denotes the position update of other dung beetles:

7 $$x_{i} \left( {t + 1} \right) = x_{i} \left( t \right) + C_{1} \times \left( {x_{i} \left( t \right) - Lb^{b} } \right) + C_{2} \times \left( {x_{i} \left( t \right) - Ub^{b} } \right)$$

Some dung beetles steal dung balls from other dung beetles, i.e., dung beetles engage in theft, while their locations are constantly updated:

8 $$x_{i} \left( {t + 1} \right) = X^{b} + S \times g \times \left( {\left| {x_{i} \left( t \right) - X^{*} } \right| + \left| {x_{i} \left( t \right) - X^{b} } \right|} \right)$$

where $g$ is a random variable obeying a normal distribution and $s$ is constant.

In summary, DBO simulates the complex behavior process of dung beetles^34^ and continuously learns and adjusts hyperparameters in deep learning optimization, achieving global optimization and ultimately finding the optimal position^33^.

### Predictive model performance analysis metrics

In the domain of machine learning, a subfield of artificial intelligence, the metrics MSE, RMSE, MAE, MAPE, and R² are frequently utilized for evaluating model performance. This study applies these five error metrics to examine prediction errors across various models, both prior to and following optimization. MSE quantifies the average of the squared differences between predicted and actual values. RMSE provides a standard measure for evaluating model prediction outcomes, indicating the extent of deviation between observed and true values. MAE represents the mean of the absolute prediction errors, while MAPE is a statistical measure that describes prediction accuracy. For these four error metrics, lower values indicate superior predictive performance. R² assesses a model’s capacity to account for variability in the output variables based on input variables; values approaching 1 signify enhanced explanatory power and predictive capability. The mathematical expressions for these five metrics are presented as follows:

9 $$MSE = \frac{1}{{\text{n}}}\sum\limits_{{i = 1}}^{n} {\left( {\hat{y}_{i} - y_{i} } \right)} ^{2}$$

10 $$RMSE = \sqrt {\frac{1}{n}\sum\limits_{{i = 1}}^{n} {\left( {y_{i} - \hat{y}_{i} } \right)^{2} } }$$

11 $$MAE\left( {y,\hat{y}} \right) = \frac{1}{n}\sum\limits_{{i = 1}}^{n} {\left| {y_{i} - \hat{y}_{i} } \right|}$$

12 $$MAPE = \frac{1}{n}\sum\limits_{{i = 1}}^{n} {\left| {\frac{{y_{i} - \hat{y}_{i} }}{{y_{i} }}} \right|}$$

13 $$R^{2} = 1 - {{\sum\nolimits_{{i = 1}}^{n} {\left( {y_{i} - \hat{y}_{i} } \right)} ^{2} } \mathord{\left/ {\vphantom {{\sum\nolimits_{{i = 1}}^{n} {\left( {y_{i} - \hat{y}_{i} } \right)} ^{2} } {\left( {\sum\nolimits_{{i = 1}}^{n} {\left( {y_{i} - \bar{\hat{y}}} \right)^{2} } } \right)}}} \right. \kern-\nulldelimiterspace} {\left( {\sum\nolimits_{{i = 1}}^{n} {\left( {y_{i} - \bar{\hat{y}}} \right)^{2} } } \right)}}$$

${y_i}$: ZTD values (true/observed), ${\bar {y}_i}$: model ZTD value (predicted), $\hat {y}$: mean ZTD value,$i$: 1,2, … ,n; $i$: calendar element

### Data preprocessing

This paper conducts a comprehensive evaluation of the model’s forecasting performance, both overall and at the individual level. The overall performance is assessed after training the model on data from 40 Train_Stations on a monthly basis, while the individual performance is evaluated following training on 6 Feature_Stations within the dataset. To enhance model performance and ensure the accuracy of analytical outcomes, the study undertakes preprocessing of the raw data. This preprocessing includes the imputation of missing values and normalization, aimed at improving data quality and the effectiveness of the analysis. Specifically, cubic spline interpolation is employed to address missing values, utilizing cubic curves between known data points. This method is preferred over quadratic interpolation due to its superior smoothness and flexibility, which make it more suitable for complex data types. For data normalization, the minimum value scaling method is applied, as detailed in Formula (12). The normalization process is achieved by calculating the ratio of the difference between X and X_MIN_ to the difference between X_MAX_ and X_MIN_.

14 $$X^{\prime} = \left( {X - X_{{MAX}} } \right)/\left( {X_{{MAX}} - X_{{MIN}} } \right)$$

## Experimental analysis

###  Comparative analysis of the prediction outcomes between the ZTD-CLMA model and the ZTD-DBO-CLMA model

To assess the predictive efficacy of the ZTD-CLMA and ZTD-DBO-CLMA models, we conducted an analysis of ZTD forecasts across 40 train stations over a continuous five-month period. The ZTD-CLMA model served as the baseline, upon which deep learning optimization was performed using DBO, utilizing the Adam optimizer over 150 epochs. In this investigation, 90% of the dataset was allocated for ZTD model training, while the remaining 10% was reserved for ZTD prediction, evaluated at monthly intervals. The results indicated that the ZTD-CLMA model exhibited commendable predictive performance, whereas the optimized ZTD-DBO-CLMA model achieved superior accuracy in ZTD predictions.

**Fig. 3:**
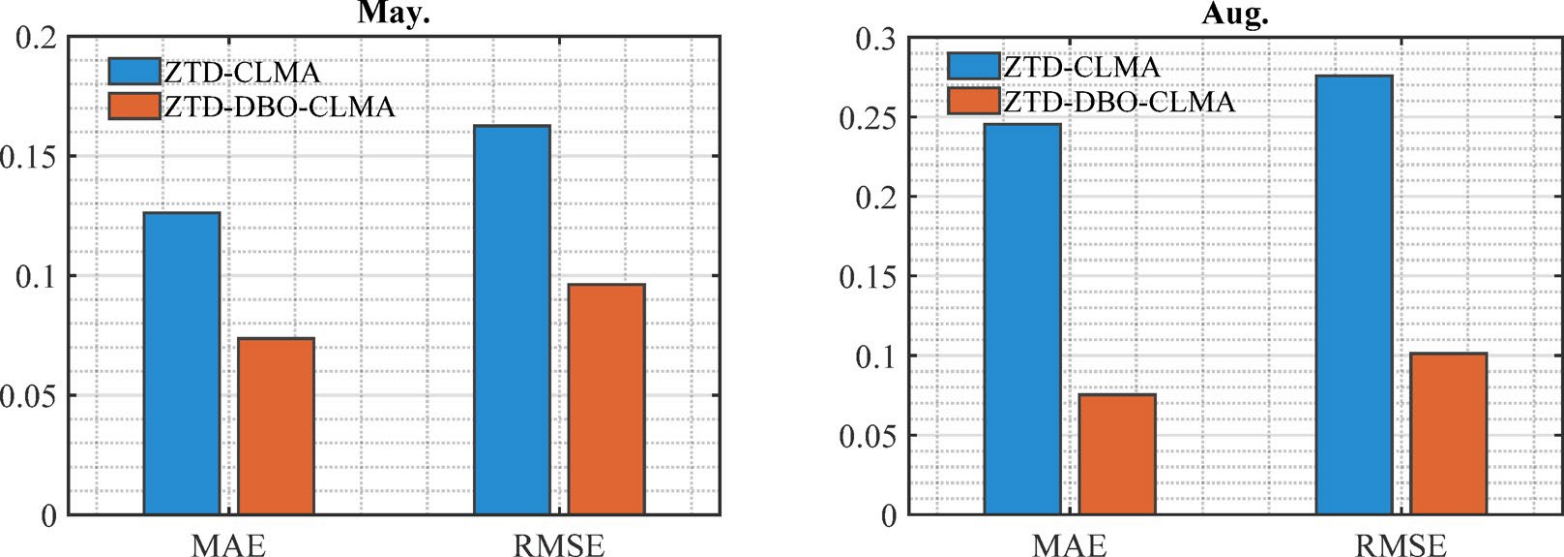
A comparative analysis of the MAE and RMSE between the ZTD-CLMA and ZTD-DBO-CLMA models is presented. The bar chart illustrates the findings, with the blue bars indicating the performance of the ZTD-CLMA model and the red bars depicting the outcomes of the ZTD-DBO-CLMA model.

In evaluating model performance, this study prioritized the analysis of MAE and RMSE metrics. Due to space limitations, data from May and August were selected as representative examples to illustrate the relevant prediction outcomes. Figure 3 presents a comparative analysis of MAE and RMSE errors between the ZTD-CLMA and ZTD-DBO-CLMA models. The prediction results for May were deemed satisfactory, with the ZTD-CLMA model yielding MAE and RMSE values of 1.26 mm and 1.63 mm, respectively, while the ZTD-DBO-CLMA model exhibited MAE and RMSE values of 0.74 mm and 0.96 mm, respectively. The results demonstrate that the integration of CNN and LSTM models markedly enhances prediction accuracy across both temporal and spatial dimensions. In August, a notable disparity in prediction errors was observed: the ZTD-CLMA model exhibited a MAE of 2.45 mm, whereas the ZTD-DBO-CLMA model achieved a substantially lower MAE of 0.75 mm. This suggests that the latter model attains higher prediction accuracy following DBO optimization. Furthermore, the RMSE for the ZTD-CLMA model was 2.76 mm, compared to only 1.01 mm for the ZTD-DBO-CLMA model. A comparative analysis of the prediction errors reveals that both the MAE and RMSE values for the ZTD-DBO-CLMA model are consistently lower than those for the ZTD-CLMA model, thereby underscoring the superior predictive performance of the ZTD-DBO-CLMA model and corroborating its enhanced accuracy^35^.

**Fig. 4:**
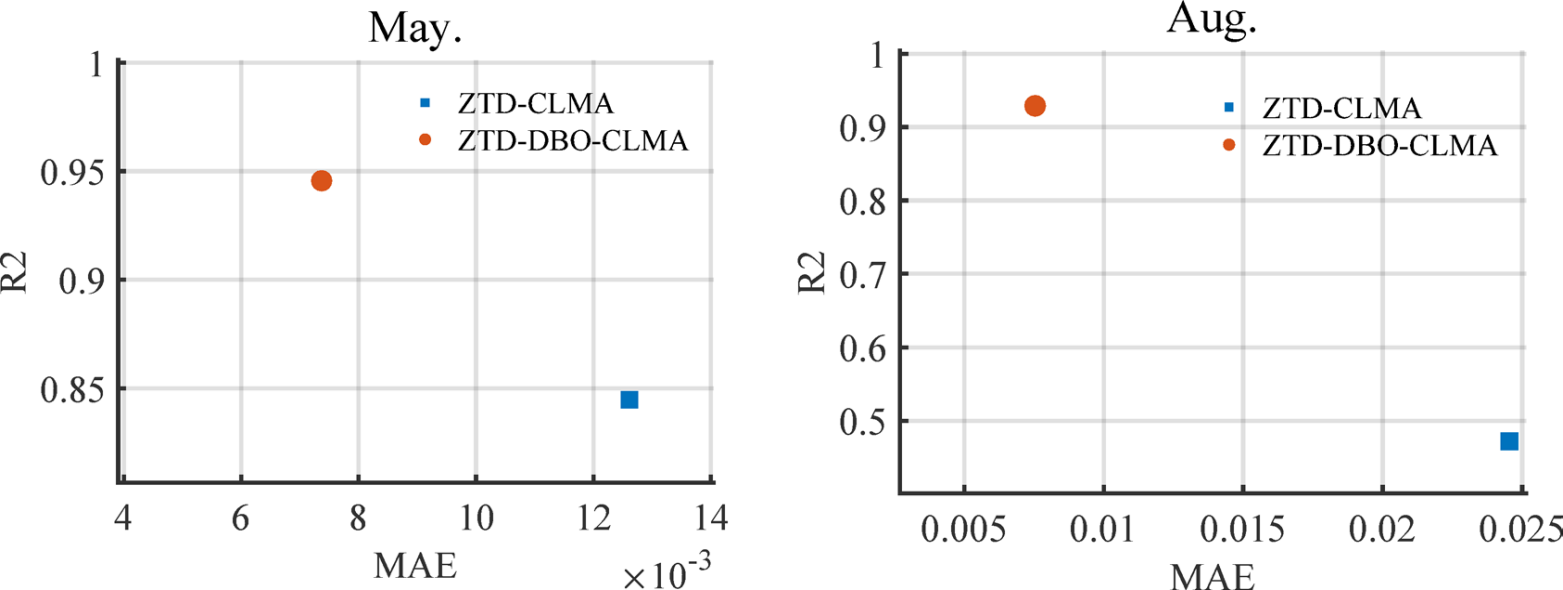
Comparison of R2 and MAE errors between ZTD-CLMA and ZTD-DBO-CLMA models.

To effectively demonstrate the predictive performance and high efficiency of the ZTD-DBO-CLMA model, and to address issues related to gradient vanishing or explosion during modeling, this study incorporates R² evaluation into the analysis. This approach allows for a comprehensive assessment of model performance by integrating R² values with MAE values. Fig. 4 illustrates the two-dimensional point distribution of the combined R² and MAE errors for both the ZTD-CLMA and ZTD-DBO-CLMA models in May and August. As depicted in the figure, the ZTD-DBO-CLMA model exhibits a high level of goodness of fit, while its MAE values are significantly lower than those of the ZTD-CLMA model. A thorough comparison in both vertical and horizontal dimensions reveals that the ZTD-DBO-CLMA model demonstrates superior performance in terms of R² and MAE.

**Fig. 5:**
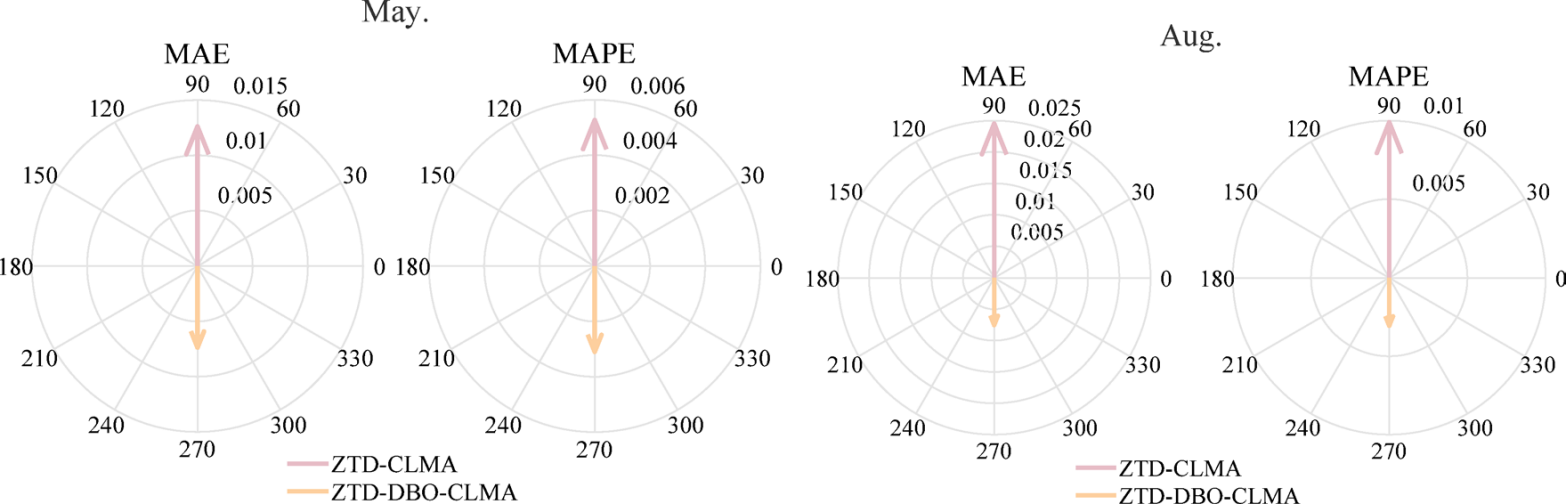
Double-loop comparison diagram of error values between ZTD-CLMA and ZTD-DBO-CLMA.

From the standpoint of predictive model accuracy, this study performs a synchronous analysis of the MAE and MAPE metrics, both of which serve as effective indicators of the predictive performance of the ZTD model. Figure 5 presents a comparative analysis of the MAE and MAPE values for the ZTD-CLMA and ZTD-DBO-CLMA models during the months of May and August. The relative radii in the double-ring comparison diagram distinctly depict the magnitude and variation of error values for these months. The pink upward-pointing arrows denote the MAE and MAPE values for the ZTD-CLMA model, with MAE values recorded at 1.26 mm and 2.45 mm, and MAPE values at 0.53% and 0.99%, respectively. Conversely, the orange downward-pointing arrows, which are shorter, represent the error values for the ZTD-DBO-CLMA model optimized by the DBO algorithm, with MAE values of 0.74 mm and 0.75 mm, and MAPE values consistently at 0.31%. These evaluation results underscore the efficacy of the model optimization, demonstrating that the DBO-optimized ZTD-DBO-CLMA model exhibits enhanced stability, convergence, and substantial generalization capabilities.

**Fig. 6:**
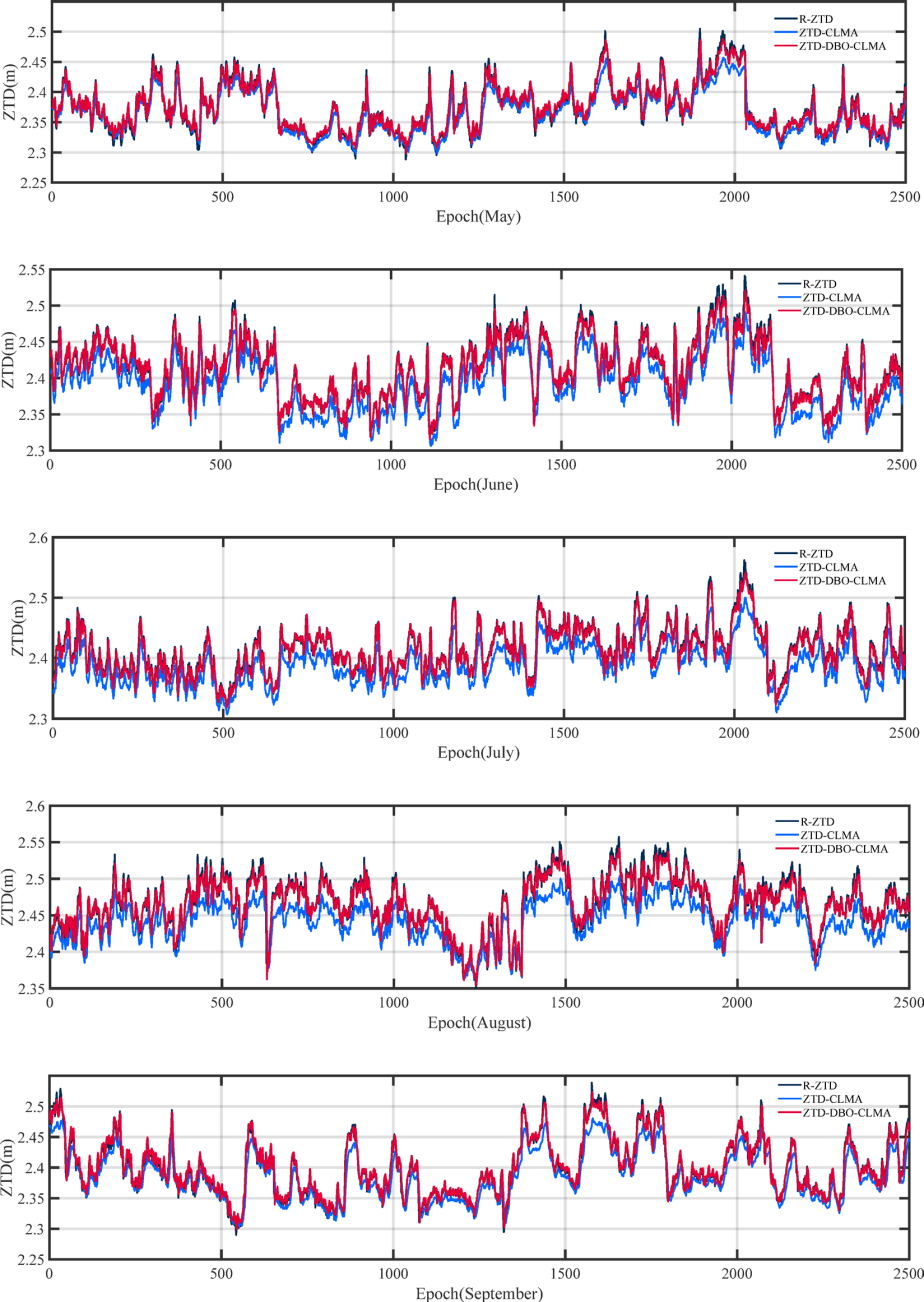
A comparative analysis of the prediction outcomes of the R-ZTD model versus the ZTD-CLMA and ZTD-DBO-CLMA models is conducted. The R-ZTD model serves as the benchmark, against which the predictive performance of the ZTD-CLMA and ZTD-DBO-CLMA models is evaluated.

The distribution of land and sea areas across Europe exhibits significant variability, and the predicted values from various ZTD models also demonstrate notable discrepancies. Furthermore, differences in the influence of natural conditions, such as temperature and precipitation, are evident. Figure 6 presents a comparison of the prediction results from the R-ZTD, ZTD-CLMA, and ZTD-DBO-CLMA models for 40 train stations over a period of five consecutive months. This figure illustrates that the predictions for May achieved the anticipated optimal outcomes, with the ZTD-CLMA and ZTD-DBO-CLMA model predictions aligning more closely with the actual values. From the perspective of data and modeling, two prominent advantages are apparent: firstly, the quality of the ZTD data product for this month is exceptionally high; secondly, the model itself exhibits stable performance and strong adaptability. Similarly, in September, the predicted values oscillated around the actual values. Notably, the prediction performance of the ZTD-DBO-CLMA model, optimized through DBO, is significantly superior to that of the ZTD-CLMA model, which lacks hyperparameter tuning. It is noteworthy that the predictive performance of both models was suboptimal from June to August, with the ZTD-CLMA model displaying considerable variability. This outcome may be attributed to the pronounced land-sea contrasts prevalent during this period. Moreover, June to August coincides with the rainy season, during which fluctuations in precipitation and temperature may exert a substantial impact. In contrast, the ZTD-DBO-CLMA model closely aligns with the actual values, thereby demonstrating exceptional stability. Additionally, the ZTD-DBO-CLMA model maintains high stability throughout the selected annual accumulation period, further underscoring the superior performance of the DBO-optimized CNN and LSTM fusion multi-head attention mechanism.

**Fig. 7:**
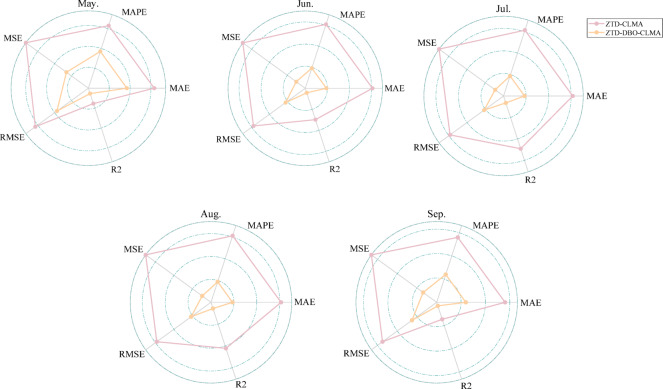
The ZTD-CLMA and ZTD-DBO-CLMA models underwent a thorough evaluation utilizing five distinct assessment indicators. The results of this dual model evaluation for the European region, covering the months of May through September 2022, are presented sequentially from left to right and top to bottom.

To assess the overall performance of the ZTD prediction model, this study employs a balanced consideration of five evaluation metrics. Figure 7 illustrates the comprehensive evaluation framework encompassing these metrics for the model. The five concentric circles correspond to five consecutive months of evaluation. The pink dotted line, along with the inner and outer rings of the circle, represents the aggregated evaluation outcomes of the five metrics for the ZTD-CLMA model. In contrast, the orange-yellow dotted line and the innermost ring of the circle depict the aggregated evaluation outcomes for the ZTD-DBO-CLMA model. The differences in predictive performance between the two models are both evident and substantial, with the ZTD-DBO-CLMA model exhibiting superior overall performance. While the ZTD-CLMA model, lacking DBO optimization, performs satisfactorily, it displays a marked improvement in various performance evaluations following DBO optimization. This indicates that DBO effectively optimizes model parameters, thereby enhancing model quality.

This study evaluated the goodness of fit, with R² values in the validation results ranging from 0 to 1, where values approaching 1 indicate a superior fit. The R² values for the ZTD-DBO-CLMA model all exceeded 90%, approaching the ideal value of 1, thereby demonstrating that the optimized model exhibits a high degree of goodness of fit. Table 3 provides a detailed account of the specific error values from May to September 2022.

**Table 3:** Evaluation index values for the ZTD-CLMA and ZTD-DBO-CLMA models.

MOUTH	ERROR	ZTD-CLMA	ZTD-DBO-CLMA
May	RMSE(mm)	1.63	0.96
	MAE(mm)	1.26	0.74
	MAPE(%)	0.53	0.31
	R^2^(%)	84.49	94.56
June	RMSE(mm)	2.52	0.96
	MAE(mm)	2.30	0.72
	MAPE(%)	0.95	0.30
	R^2^(%)	62.71	94.61
July	RMSE(mm)	2.91	1.06
	MAE(mm)	2.67	0.81
	MAPE(%)	1.10	0.33
	R^2^(%)	38.51	91.93
August	RMSE(mm)	2.76	1.01
	MAE(mm)	2.45	0.75
	MAPE(%)	0.99	0.31
	R^2^(%)	47.21	92.90
September	RMSE(mm)	1.97	0.90
	MAE(mm)	1.58	0.67
	MAPE(%)	0.65	0.28
	R^2^(%)	81.72	96.20

The table presented above demonstrates that the optimized model achieves high-precision predictions while maintaining superior fitting performance. The comparative results further substantiate the superiority and exceptional performance of the model proposed in this study.

### ZTD-DBO-CLMA model performance evaluation

**Fig. 8:**
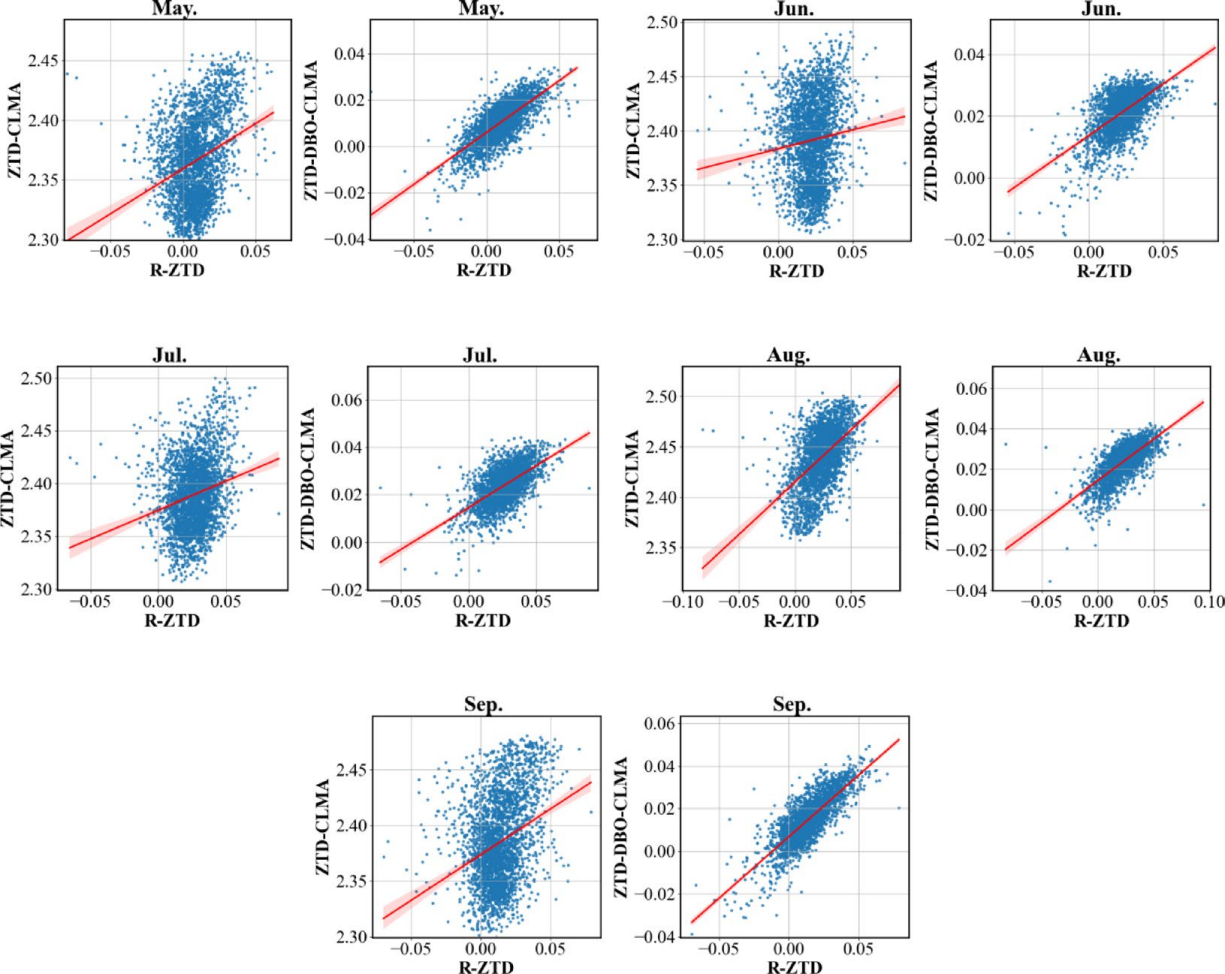
A comparative analysis of residual plots was conducted between the R-ZTD and ZTD-CLMA, as well as the ZTD-DBO-CLMA regression models. The analysis encompassed data from the months of May through September, organized in pairs. In the plots, blue dots denote the residual data points, while red lines indicate the normalized lines, and red shading illustrates the confidence intervals.

To thoroughly assess the performance of the ZTD-DBO-CLMA model, this study undertook an extensive comparison of the prediction outcomes for the months of May, June, July, August, and September. Residual plots and regression analyses, as depicted in Fig. 8, were employed to elucidate the discrepancies between the prediction results of the two models. By examining the residual plots, comparisons were made between the predicted values of both the ZTD-CLMA model and the ZTD-DBO-CLMA model against the actual values. The residuals in the plots on the right exhibit greater concentration, indicating stronger correlations among data points and a discernible directional trend in their distribution, thereby suggesting enhanced model stability. From May to September, encompassing the summer and autumn seasons, the European region experiences notable land-sea distribution, characterized by significant and intense variations in water vapor. These changes are further influenced by the monsoon climate, leading to increasingly complex environmental conditions. Within this context, the ZTD-DBO-CLMA model, enhanced through DBO optimization, exhibits remarkable stability and adaptability. It effectively addresses the challenges of local optima while maintaining high predictive accuracy, thereby underscoring the efficacy of the DBO algorithm in hyperparameter optimization.

### Test validation based on different land and sea distribution sites

In light of the unique natural geographical characteristics and the uneven distribution of sites, six Feature_Stations within the region were selected for testing and verification in the test results. Table 4 provides detailed information on these six Feature_Stations, including comprehensive numerical values pertaining to the spatial domain.

**Table 4:** The dataset provides comprehensive information regarding the regional feature_station, encompassing its name, geographical coordinates (longitude and latitude), and elevation above sea level.

	City	Station Name	Latitude(°)	Longitude(°)	Height Of Station Above Sea Level(m)
1	Frankfurt/Main	FFMJ	50.09058	8.66497	130.14323
2	Graz	GRAZ	47.06713	15.493481	490.81055
3	Matera	MATE	40.64913	16.70470	490.13930
4	Riga	RIGA	56.94862	24.05877	13.58371
5	Lviv	SULP	49.83559	24.01449	339.62753
6	Visby	VIS0	57.65387	18.36732	54.76146

**Fig. 9:**
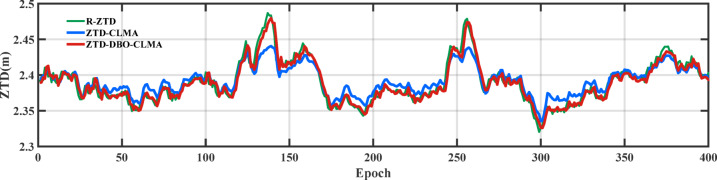
Test R-ZTD, ZTD-CLMA, ZTD-DBO-CLMA model result plots.

To provide a clearer representation of the accuracy of the two models in validating Feature_Station, the prediction outcomes for six Feature_Stations were visualized. Figure 9 illustrates three lines that depict the variations in the R-ZTD true values, the ZTD-CLMA model predictions, and the ZTD predictions from the ZTD-DBO-CLMA model, which has been optimized using DBO, across the six Feature_Stations.

**Fig. 10:**
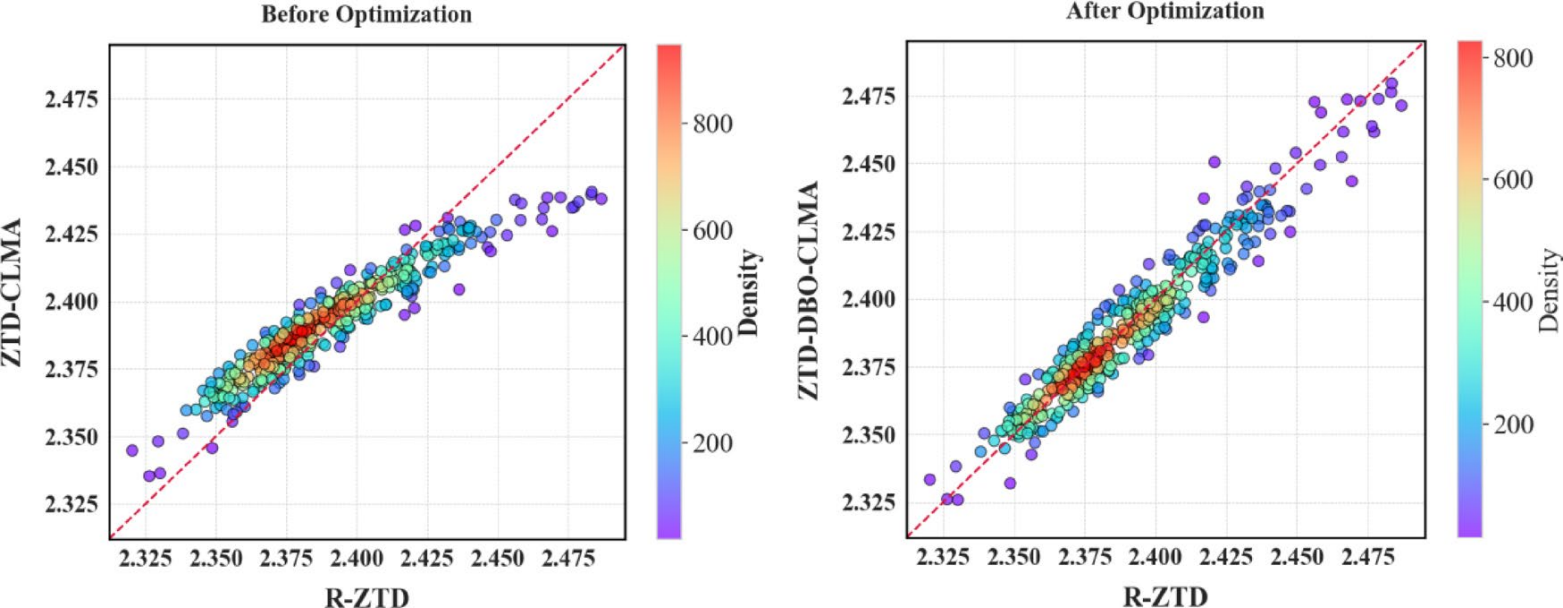
Please review the correlation comparison analysis chart for the Feature_Stations. The six Feature_Stations under consideration are FFMJ, GRAZ, MATE, VIS0, SULP, and RIGA. The chart utilizes the reference value R-ZTD on the horizontal axis, while the vertical axes represent the ZTD-CLMA model prediction value and the ZTD-DBO-CLMA model prediction value.

Following comparative validation, the variations among the three models demonstrate a correlated trend. However, from an error analysis standpoint, although the trend of the ZTD-CLMA model aligns with the other two models, it exhibits a disadvantage at prominent peaks or troughs, resulting in significant deviations in ZTD prediction values. Conversely, the ZTD-DBO-CLMA model, optimized through DBO, effectively addresses this limitation. Whether encountering scenarios with large fluctuations in ZTD values or periods of stable changes, the ZTD-DBO-CLMA model consistently achieves high-precision predictions, producing optimal results. The subsequent sections will provide a comprehensive description of the comparison and validation process. To ensure the validity of the validation results, this study employed six Feature_Station data points for performance evaluation of the ZTD-DBO-CLMA model, with the visualization results presented below (Fig. 10).

As illustrated in the preceding figure, the overall distribution trend without DBO optimization exhibits a noticeable skewness, with residual points not uniformly distributed around the regression line. This observation suggests that the predictive performance of the ZTD-CLMA model is suboptimal. Within the range of 2.425 m to 2.475 m, the distribution of ZTD values demonstrates a bias toward the R-ZTD side, potentially influenced by the geographical distribution of land and sea. While the distribution of the six Feature_Stations appears relatively uniform, certain Feature_Stations situated closer to the ocean may be affected by the European monsoon or land-sea interactions, resulting in significant discrepancies in the distribution plot. In contrast to the residual regression distribution map on the left, the residuals on the right are more concentrated along the regression line and exhibit a more uniform distribution on both sides of the line. Therefore, it can be inferred that the ZTD-DBO-CLMA model, when optimized by DBO, remains largely unaffected by significant errors arising from environmental variations. Building on the preceding discussion, this paper undertakes a comprehensive examination of the correlation. Initially, the study investigates the trend of changes between the predicted values of the ZTD-DBO-CLMA model and the reference R-ZTD values, revealing a high degree of correlation between the two. Subsequently, the residual plot illustrates the prediction outcomes for six distinct Feature_Station sites, with all general sites consistently distributed on both sides of the regression line. Each data point comprises both the R-ZTD value and the ZTD-DBO-CLMA predicted value, underscoring a robust correlation between the reference and predicted values. Different colored rings denote varying densities of data distribution. According to the bar chart on the right, higher values denote a greater density of data points, with the color spectrum transitioning from purple to red, where a darker red signifies more densely distributed data points. Notably, within the ZTD value range of 2.35 m to 2.40 m, the most densely populated red data points are consistently aligned along the regression line, clearly illustrating the correlation trend between the variables. Furthermore, the absence of significant outliers in the figure can be attributed to the high quality and precision of the data sources, as well as the comprehensive preprocessing conducted prior to the experiment. This ensures the accuracy and efficiency of the ZTD-DBO-CLMA model’s predictive outcomes. In summary, Pearson correlation coefficient analysis was employed in this study, and the data indicate that the experimental results used for modeling are consistent. These findings underscore the correlation between the reference values and the ZTD-DBO-CLMA model’s predicted values, thereby establishing a theoretical foundation for subsequent analysis.

**Fig. 11:**
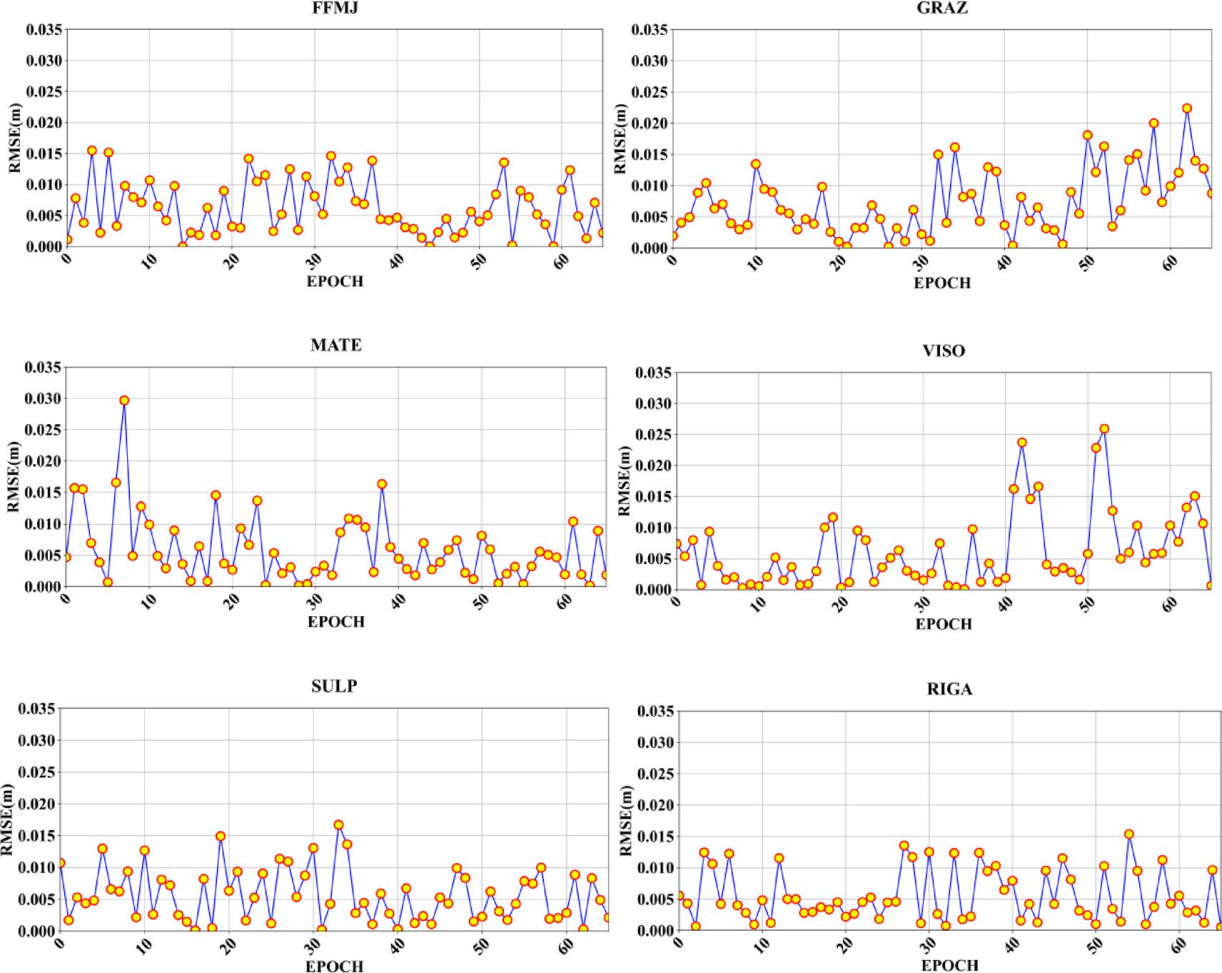
Evaluate the RMSE for the six Feature Stations by plotting the epoch on the x-axis and the corresponding RMSE values on the y-axis.

This study examines the variation in prediction errors of the ZTD-DBO-CLMA model by analyzing the correlation between its predicted values and the reference values. Figure 11 presents the experimental results of the RMSE comparison across six Feature Stations. As depicted in the figure, the fluctuations are generally consistent, with RMSE values ranging from 0 to 0.015 m, although some individual error values slightly exceed 0.015 m. Given that the deep learning model employed in this research is based on a large dataset, the influence of these errors is considered negligible.

A comprehensive analysis of the RMSE for each Feature_Station reveals the following insights: Firstly, the VISO validation point is uniquely situated, being surrounded by the sea, and is significantly influenced by the sea-land monsoon. As depicted in Fig. 11, there are distinct and continuous jump points, which are likely attributable to its unique geographical location and the pronounced impact of sea-land distribution differences. Similarly, the error map for the MATE Feature_Station exhibits several jump points, which may be due to its proximity to the ocean, the uneven distribution of surrounding stations, and potential influences from monsoons and temperature variations. In the error analysis map for the GRAZFeature_Station, the error remains consistently low during the initial half of the time series; however, a gradual increase in error is evident in the latter half. As illustrated in Fig. 1 of this paper, the surrounding stations are evenly distributed, yet there is a notable absence of stations in the southeast direction of Feature_Station. This suggests that the observed phenomenon may be significantly influenced by the uneven distribution of stations. Apart from the southeast direction, the remaining stations are densely and evenly distributed, with no apparent discontinuities, which aligns with the data point distribution depicted in the error analysis map. When compared to the other three Feature_Station validations, the RMSE values for FFMJ, SULP, and RIGAFeature_Station consistently demonstrate favorable performance, with error values generally below or near 0.015 m and no significant outliers, indicating relatively stable data points. Notably, the FFMJ Feature_Station is situated within the continent, surrounded by uniformly distributed stations, and is largely unaffected by variations in the European land-sea distribution. The RMSE accuracy at this station is consistently below 0.015 m, with uniform fluctuations in data points, leading to precise and stable prediction outcomes, thereby underscoring the stability and reliability of the ZTD-DBO-CLMA model. Despite RIGAFeature_Station’s location at the boundary between land and sea and the absence of nearby stations, its RMSE remains relatively stable, with no significant outliers and unaffected by the distribution of surrounding stations or land-sea distribution differences. Similarly, SULPFeature_Station, located inland, experiences minimal environmental influence, resulting in smaller error fluctuations. The error analysis diagram corroborates the high prediction accuracy at this station.

### Test validation under different weather conditions

To enhance the robustness of test validation, this study conducts a comparative analysis under varying weather conditions, including sunny, rainy, and extreme weather scenarios. Weather type thresholds are established based on the ZTD values. Data from May 2022 are utilized as validation data. Specifically, a ZTD value within the range of 2.000 to 2.200 m is classified as sunny weather, values between 2.200 and 2.400 m are categorized as rainy weather, and ZTD values exceeding 2.400 m are identified as extreme weather conditions.

**Fig. 12:**
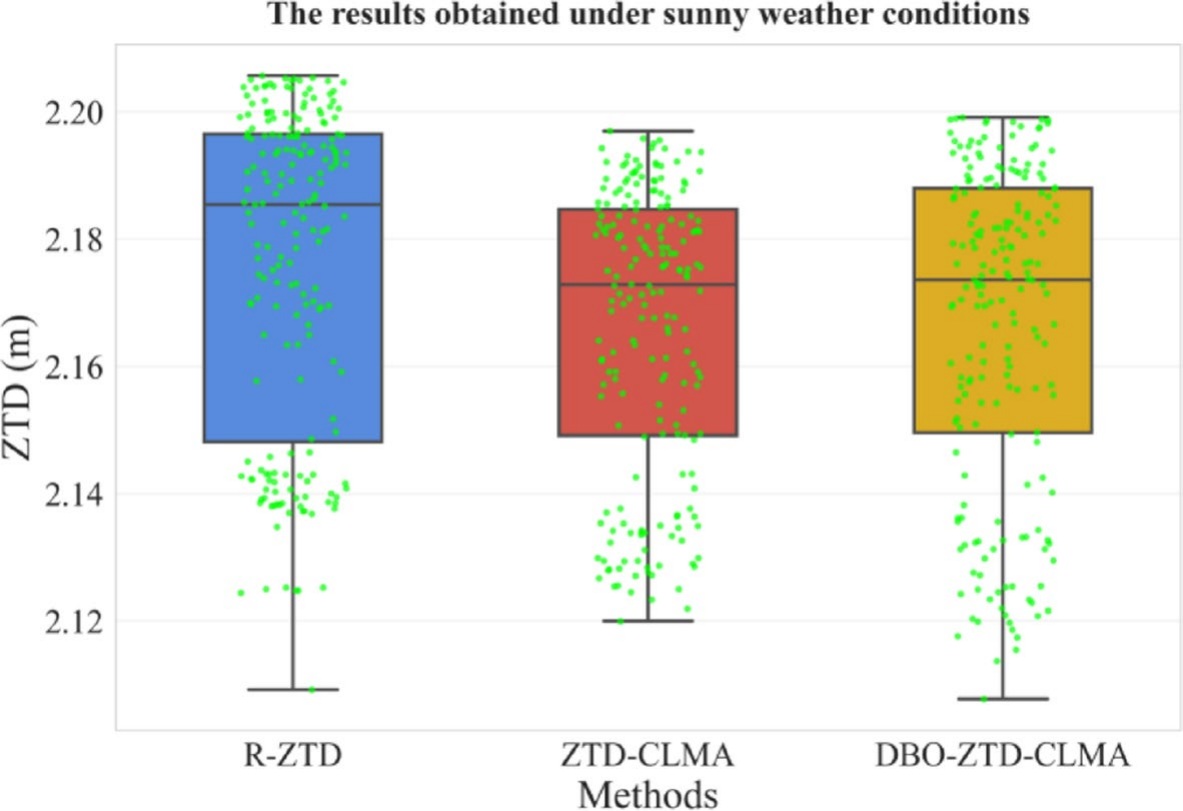
Results under clear weather conditions.

In sunny weather, comparative analysis reveals that the box plot of the DBO-ZTD-CLMA model, optimized by DBO, more closely resembles that of the R-ZTD model, as depicted in Fig. 12. Additionally, the median line of the DBO-ZTD-CLMA model’s box plot is higher than that of the ZTD-CLMA model, indicating superior performance. Under clear weather conditions, the DBO-ZTD-CLMA model exhibits significantly enhanced stability, corroborating the model performance results discussed in the preceding subsection.

**Fig. 13:**
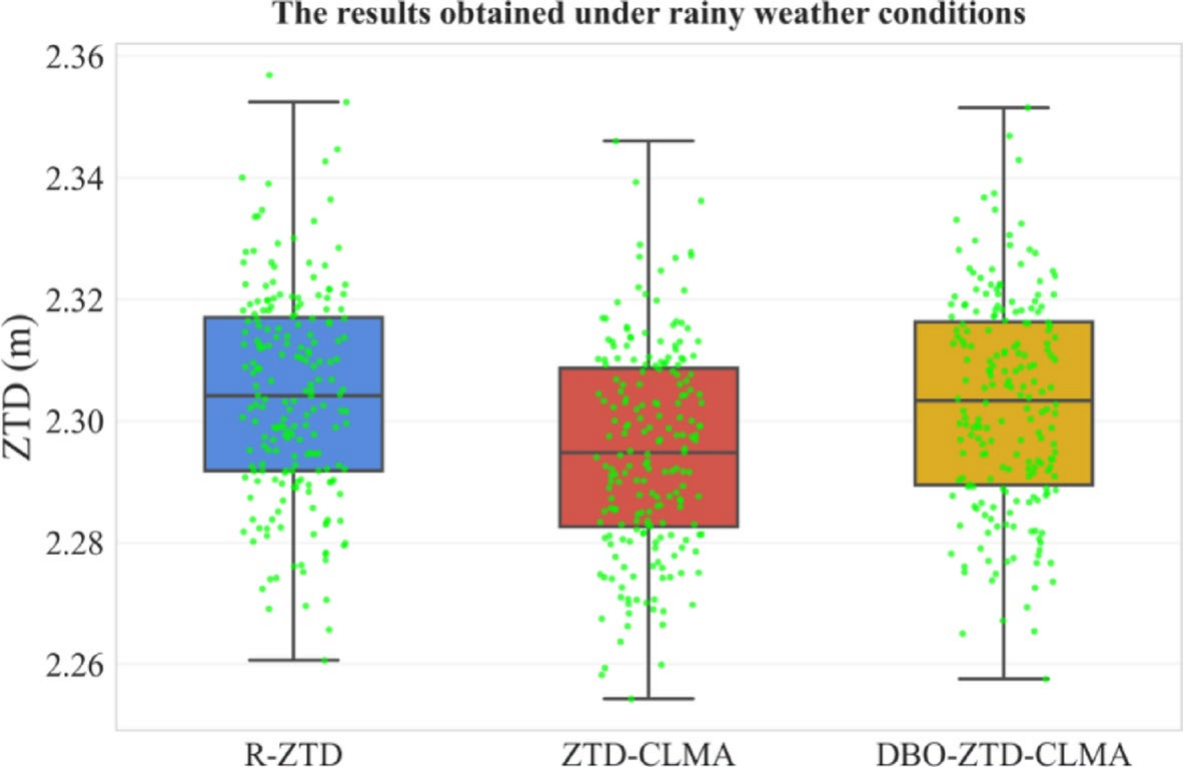
Results under rainy weather conditions.

Rainy Weather: In a similar vein, this study conducted a validation analysis for rainy weather conditions, with Fig. 13 illustrating the model’s performance. The DBO-optimized DBO-ZTD-CLMA model demonstrates superior performance compared to the ZTD-CLMA model, aligning with the validation results presented in the preceding subsection.

**Fig. 14:**
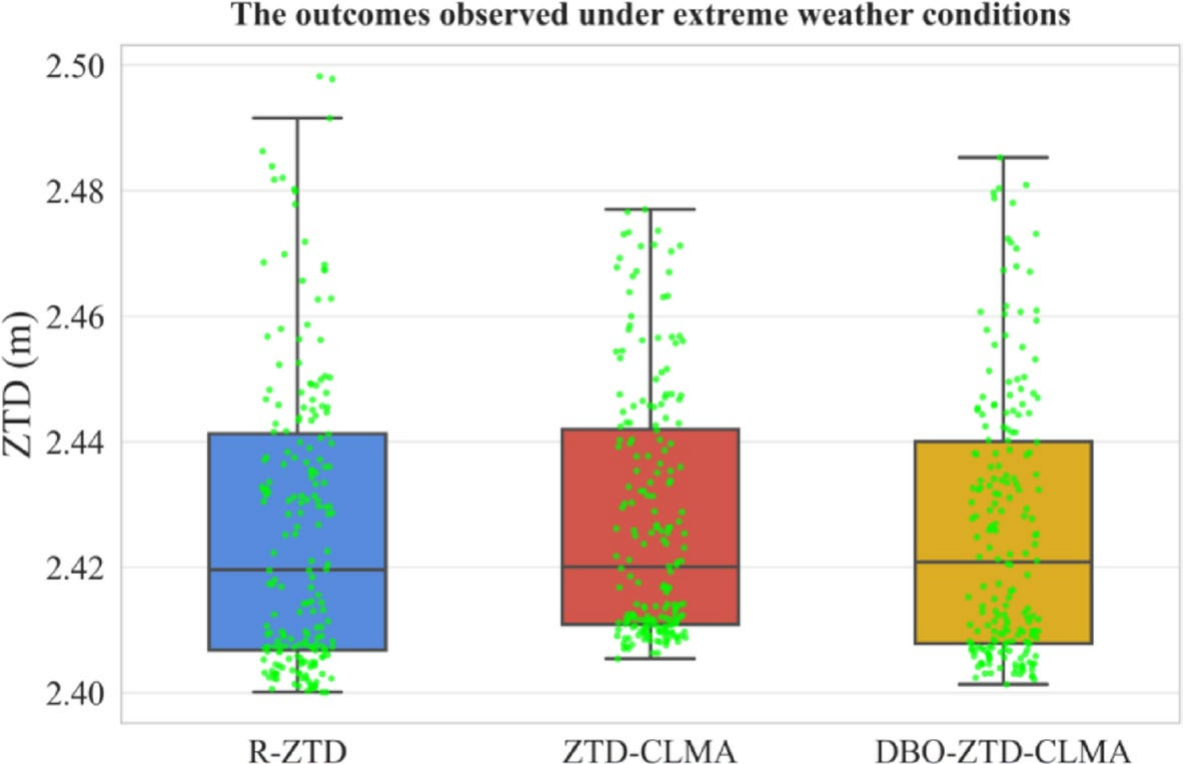
Results under extreme weather conditions.

Extreme Weather Conditions: To enhance the generalizability and persuasiveness of the validation results, this study visualized the model’s predictive performance under extreme weather conditions, as depicted in Fig. 14. The DBO-ZTD-CLMA model exhibited exceptional performance. Upon comparing the three models, the yellow and blue box plots display greater similarity in terms of box height and shape, with a higher median line. Consequently, it can be inferred that the model optimized by DBO outperforms the others, corroborating the validation results from the previous subsection.

**Fig. 15:**
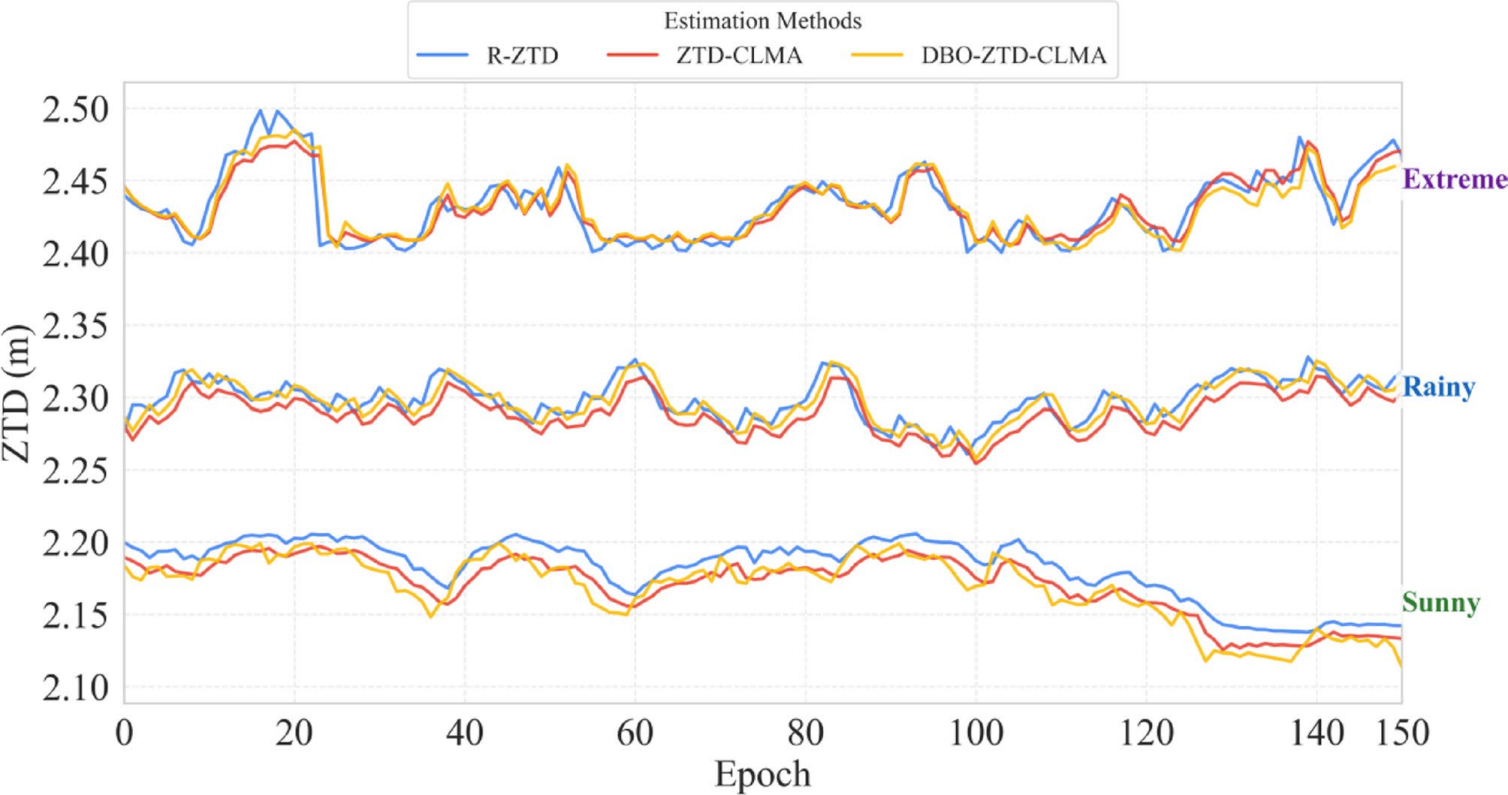
Comparison results under sunny, rainy, and extreme weather conditions.

Based on the preceding results, further analysis is warranted. The validation dataset for this study comprises 150 sample sets collected under sunny, rainy, and extreme weather conditions, as illustrated in Fig. 15. Under sunny conditions, the DBO-ZTD-CLMA model closely aligns with the R-ZTD model, demonstrating superior performance. Similarly, during rainy conditions, the predictive outcomes of the DBO-ZTD-CLMA model are more consistent with those of the R-ZTD model. However, in extreme weather conditions, the performance of both the ZTD-CLMA model and the DBO-ZTD-CLMA model is nearly equivalent. Overall, the model’s performance is comparable to that of the R-ZTD model, with no anomalies such as discrete jump points. Even in extreme weather scenarios, such as heavy rain or thunderstorms, the model exhibits robust performance, underscoring its stability under such conditions. In conclusion, the model exhibits commendable stability and robustness across clear, rainy, and extreme weather conditions. Furthermore, it is evident that the DBO-optimized model remains largely unaffected by increasingly adverse weather conditions, which is consistent with the outcomes observed during model training.

## Results and discussion

The evaluation of the forecasting performance for the 40 Train_Stations, along with the specific assessment of the 6 Feature_Stations, reveals that DBO optimization exerts a substantial impact. As detailed in Table 3, the overall forecasting performance analysis indicates that the ZTD-DBO-CLMA model, when compared to the ZTD-CLMA model without DBO optimization, exhibits improvements of 0.31 mm in MAE and 1.38 mm in RMSE, alongside a 39.43% enhancement in goodness of fit. This clearly underscores the significant enhancement in predictive accuracy afforded by DBO optimization. Furthermore, the visualization of prediction errors at the 6 Feature_Station sites, as depicted in Fig. 11, corroborates the efficacy of DBO optimization in augmenting the accuracy and optimization outcomes of ZTD predictions. A more detailed discussion is provided below. (1) The DBO-optimized ZTD-DBO-CLMA model demonstrates enhanced adaptability in complex environmental conditions and regions with uneven station distribution. This finding corroborates the outcomes of other optimization models, such as the DBO-CNN referenced in^35^, thereby underscoring the efficacy of DBO-based optimization strategies in augmenting the model’s global search capabilities and fine-tuning hyperparameters. (2) Although the model significantly improves prediction performance for the majority of general stations, it exhibits marginally higher errors at certain stations, likely due to the intricacies of regional climate conditions. This observation aligns with the findings reported in^36^. Based on the validation comparison results from the six Feature_Stations discussed in the previous section, the ZTD-DBO-CLMA model demonstrates a robust capability to optimize hyperparameters for ZTD temporal and spatial predictions, thereby contributing to high accuracy. This renders it suitable for developing regional high-precision ZTD prediction models. It is anticipated that in PPP applications, leveraging extensive datasets for ZTD modeling will prove to be highly practical and adaptable. Cross-scenario validation reveals that the model’s errors, within the 95% confidence interval, consistently fall below the 15 mm threshold, thereby satisfying the operational application standards outlined in^37^. This stability underscores its feasibility for engineering implementation in real-time PPP solutions.

Accurate prediction of tropospheric delay necessitates the inclusion of various environmental factors. For instance, Reference^38^ focused extensively on tropospheric delay under extreme weather conditions, excluding other meteorological scenarios such as clear and rainy days from its analysis. Consequently, this study undertook comparative validation across diverse weather conditions, including clear, rainy, and extreme weather scenarios. The findings indicate that the model consistently maintains performance stability, demonstrating robust predictive capabilities irrespective of environmental severity. Furthermore, this study selected Europe as the research region, taking into full account the station density, which significantly influences prediction accuracy—a factor not considered in the analysis by Reference^36^.

The deep learning algorithm introduced in this paper, despite the hybrid architecture’s advancements in accuracy, still encounters challenges: high model complexity and computational cost, persistent bottlenecks in long sequence modeling, and areas for improvement regarding its black-box nature and convergence uncertainty. Future research could focus on reducing model complexity to decrease computational expenses. While DBO is capable of autonomously tuning parameters, the process of hyperparameter optimization can become trapped in local optima when the search space is excessively large. Furthermore, although LSTM networks mitigate the vanishing gradient issue, they demand substantial memory resources. Future research should explore algorithms with reduced spatial complexity to improve the processing of long sequences. Additionally, the random search strategy employed by DBO could benefit from integrating the DBO search path algorithm to enhance model transparency. In conclusion, the DBO-CNN-LSTM-Multihead-Attention model exhibited significant effectiveness and superior performance in the spatio-temporal sequence task within this study. Future investigations could focus on optimizing and refining this architecture with respect to memory space utilization to achieve an optimal state.

## Conclusion

This study utilizes ZTD data from 40 IGS stations across the European region over a continuous five-month period to explore high-precision modeling techniques for regional ZTD. A novel ZTD-DBO-CLMA prediction model, grounded in DBO, is introduced. Through hyperparameter optimization, this model not only enhances the efficiency of identifying optimal conditions but also ensures prediction accuracy. It effectively addresses challenges such as the uneven distribution of regional stations, inadequate optimization of data sources, and suboptimal parameter optimization effects in time series prediction. The comparative analysis of five accuracy metrics—MAE, MAPE, MSE, RMSE, and R²—across 40 IGS stations demonstrates the superior predictive performance of the ZTD-DBO-CLMA model over the ZTD-CLMA model. Further examination of six IGS stations, selected based on varying characteristics such as land-sea distribution disparities and non-uniform station distribution, along with analyses of error sequence variations, error correlation magnitudes, and RMSE values, corroborates the enhanced effectiveness and robustness of the optimized model. This ensures the accuracy, stability, and reliability of its predictive capabilities. Moreover, to substantiate the study’s credibility, data validation was conducted under diverse weather conditions, including clear, rainy, and extreme scenarios. The findings confirm that the ZTD-DBO-CLMA model consistently exhibits superior optimized performance.

The proposed ZTD-DBO-CLMA model offers a robust solution for addressing regional ZTD prediction challenges. It mitigates the influence of ZTD as an error source in GNSS precise positioning and enhances the utility of ZTD in forecasting extreme weather conditions. Future research will investigate the model’s adaptability in complex terrain environments, including its practical application in hilly and plateau regions, while also considering a wider range of geographical areas. The objective is to achieve improved model optimization and efficient performance through a relatively straightforward deep learning architecture.

## Data Availability

The data is derived from the European Center for Mesoscale Weather Forecasting , which can be downloaded from https://cds.climate.copernicus.eu/datasets.
