# Correction: Regional zenith tropospheric delay prediction using DBO-optimized CNN-LSTM with multihead attention

**DOI:** 10.1038/s41598-025-26660-3

**Published:** 2025-11-20

**Authors:** Ruixue Yang, Xu Yang, Shicheng Xie, Xuexiang Yu

**Affiliations:** 1 Key Laboratory of Aviation-Aerospace-Ground Cooperative Monitoring and Early Warning of Coal Mining-Induced Disasters of Anhui Higher Education Institutes, Anhui University of Science and Technology, KLAHEI (KLAHEI18015), Huainan, 232001 China; 2 Engineering Research Center of Mining Area Environmental and Disaster Cooperative Monitoring, Anhui University of Science and Technology, Huainan, 232001 China; 3 Urban 3D Real Scene and Intelligent Security Monitoring Joint Laboratory of Anhui Province, Huainan, 232001 China; 4 The Key Laboratory of Universities in Anhui Province for Prevention of Mine Geological Disasters, Huainan, 232001 China; 5 School of Geomatics, Anhui University of Science and Technology, Huainan, 232001 China

Correction to: *Scientific Reports* 10.1038/s41598-025-15376-z, published online 12 August 2025

The original version of this Article contained errors.

In the Data and methods section, under the subheading ‘Parallel ZTD-CLMA model for DBO optimisation’

 “Subsequently, the dataset is processed through the deep learning CNN structure layer, resulting in the formation of the CNN-LSTM-Multihead-Attention model, which enhances the model’s capability to analyze sequence data from multiple perspectives. Finally, the DBO optimization technique is applied to the ZTD-LSTM model, yielding the ZTD-DBO-LSTM model. The performance of this model is assessed using various metrics, including Mean Absolute Error (MAE), Mean Absolute Percentage Error (MAPE), Mean Squared Error (MSE), Root Mean Squared Error (RMSE), and the Coefficient of Determination (R^2^).”

now reads:

“Subsequently, the dataset is processed through the deep learning CNN structure layer, resulting in the formation of the CLMA model, which enhances the model’s capability to analyze sequence data from multiple perspectives. Finally, the DBO optimization technique is applied to the ZTD- CLMA model, yielding the ZTD-DBO- CLMA model. The performance of this model is assessed using various metrics, including Mean Absolute Error (MAE), Mean Absolute Percentage Error (MAPE), Mean Squared Error (MSE), Root Mean Squared Error (RMSE), and the Coefficient of Determination (R^2^).”

In addition, in the Experimental analysis section, under the subheading ‘Comparative analysis of the prediction outcomes between the ZTD-CLMA model and the ZTD-DBO-CLMA model’,

“The prediction results for May were deemed satisfactory, with the ZTD-CLMA model yielding MAE and RMSE values of 0.74 mm and 1.26 mm, respectively, while the ZTD-DBO-CLMA model exhibited MAE and RMSE values of 0.96 mm and 1.63 mm, respectively.”

now reads:

“The prediction results for May were deemed satisfactory, with the ZTD-CLMA model yielding MAE and RMSE values of 1.26 mm and 1.63 mm, respectively, while the ZTD-DBO-CLMA model exhibited MAE and RMSE values of 0.74 mm and 0.96 mm, respectively.”

Finally, the order of the Figures was incorrect. Figure 12 was published as Figure 15, Figure 13 was published as Figure 12, Figure 14 was published as Figure 13 and Figure 15 was published as Figure 14. The citations of the Figures in the text have been updated accordingly.

The original Figures 12, 13, 14 and 15 and their accompanying legends appear below.

**Fig. 12:**
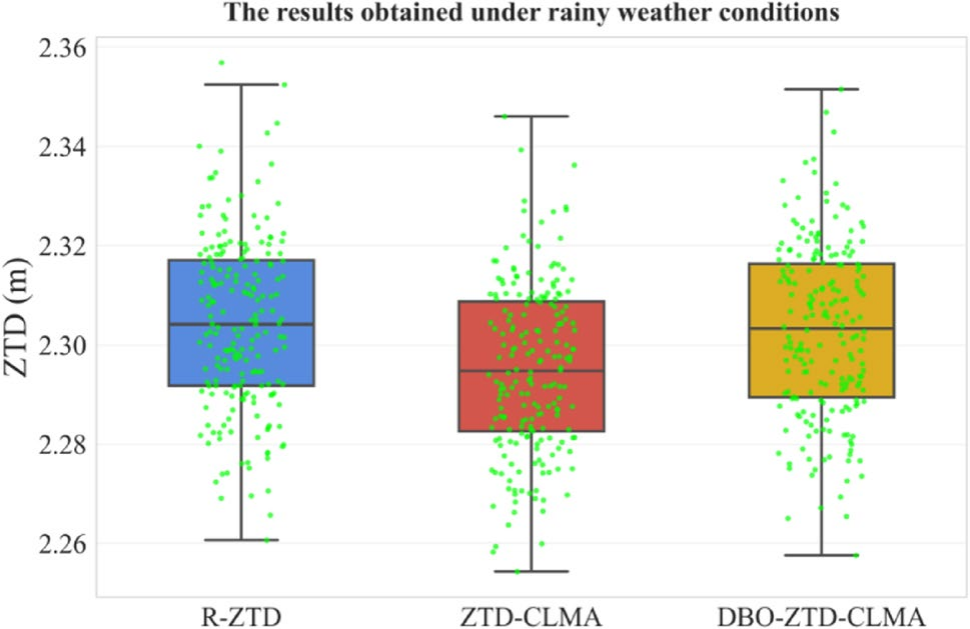
Results under rainy weather conditions.

**Fig. 13:**
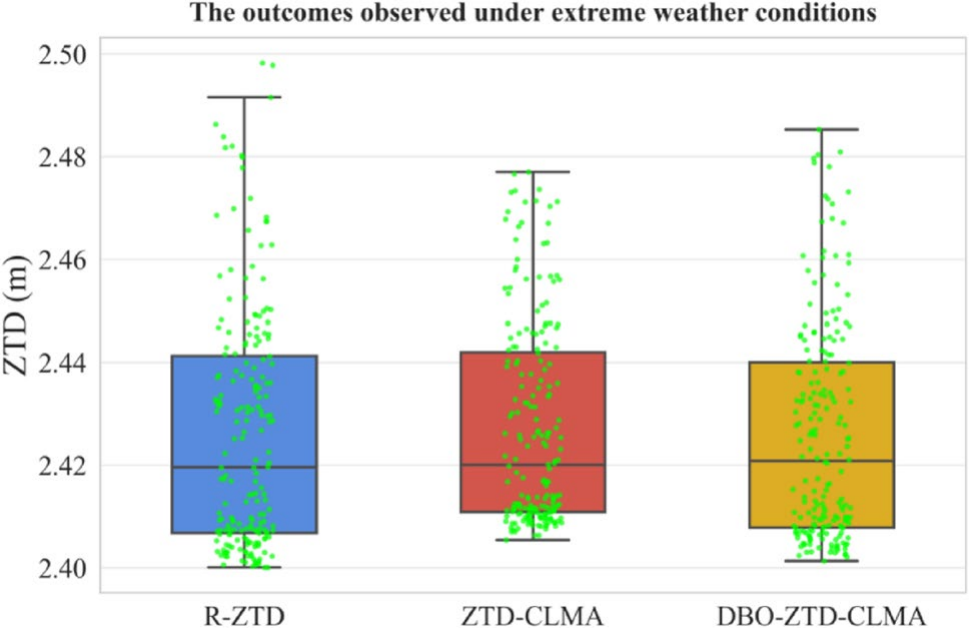
Results under extreme weather conditions.

**Fig. 14:**
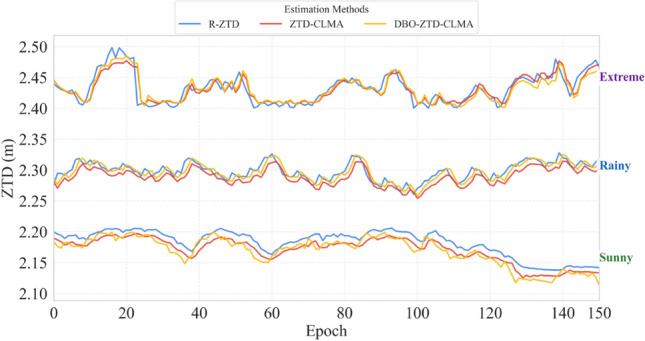
Comparison results under sunny, rainy, and extreme weather conditions.

**Fig. 15:**
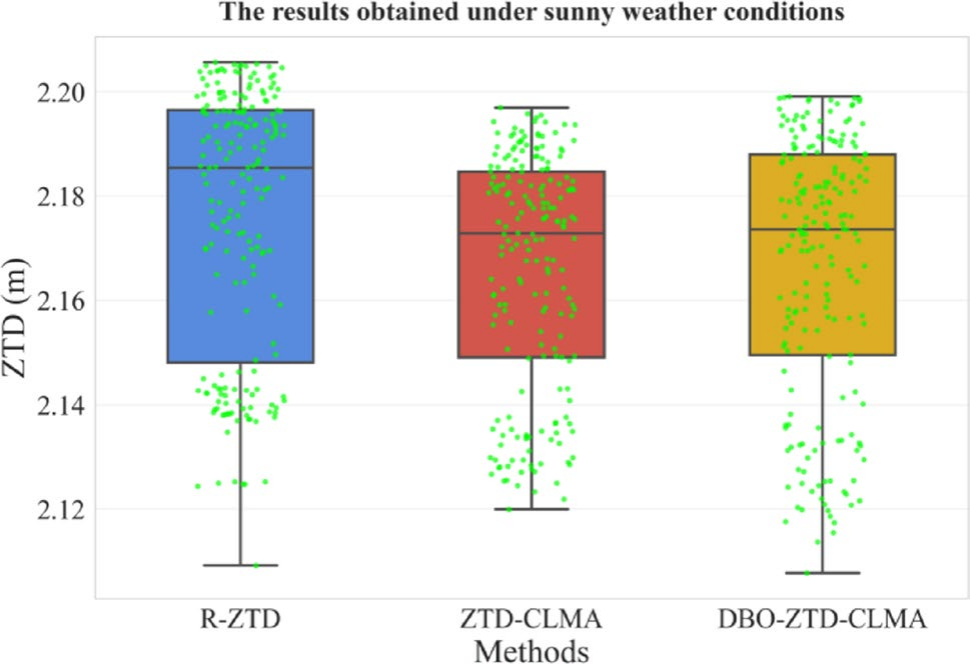
Results under clear weather conditions.

The original Article has been corrected.

